# Extraction, Structural Characterization and Anti‐Inflammatory Activity of Polysaccharides From *Conioselinum vaginatum*

**DOI:** 10.1002/fsn3.71827

**Published:** 2026-04-26

**Authors:** Conghui Ren, Yuhao Cui, Junlong Wang, Qingyou Fu, Shenghui Chen, Jie Li, Yonggang Lin

**Affiliations:** ^1^ School of Chemistry and Chemical Engineering, Key Laboratory of Yili Kazakh Medicinal Resource Exploration and Modernization Research Yili Normal University Yining P. R. China

**Keywords:** Anti‐inflammatory activity, *Conioselinum vaginatum*, Extraction, Polysaccharide, Structural characterization

## Abstract

Polysaccharides are the key functional components in *Conioselinum vaginatum*. This study aims to optimize the extraction process of *Conioselinum vaginatum* polysaccharides (CVP), investigate their structural characteristics, and evaluate their anti‐inflammatory activity. Through response surface methodology (RSM), the optimal extraction conditions were determined as follows: time 29 min, ultrasonic power 204 W, liquid–solid ratio 38:1 mL/g, and temperature 54°C. Under these conditions, the yield reached 10.12% ± 0.38%. CVP was separated and purified using a DEAE‐650‐M cellulose column and a Sephadex G‐75 gel column, yielding two homogeneous polysaccharides, CVP‐I and CVP‐II. Their molecular weights (Mw) were 26.42 ± 0.37 and 17.81 ± 0.23 kDa, respectively. Both polysaccharides were composed of varying proportions of fucose, rhamnose, arabinose, mannose, and glucose, and simultaneously contained α‐ and β‐glycosidic bonds alongside pyranose and furanose ring structures. The backbone of CVP‐I primarily consists of →4)‐Fuc*p*‐(1 → and →2,4)‐Rha*p*‐(1→, with terminal residues mainly being T‐Ara*f*. In contrast, CVP‐II is mainly composed of →2,4)‐Rha*p*‐(1 → and →4)‐Glc*p*‐(1→, with terminal residues primarily T‐Fuc*p* and T‐Ara*f*. Additionally, they exhibited different chemical compositions, stable triple‐helix conformations, low crystallinity, unique morphological characteristics, uniform particle size distribution, and excellent thermal stability. Anti‐inflammatory activity studies revealed that both CVP‐I and CVP‐II effectively regulated the production of nitric oxide (NO), prostaglandin E2 (PGE_2_), interleukin‐6 (IL‐6), interleukin‐1β (IL‐1β), and tumor necrosis factor‐α (TNF‐α) in LPS‐induced RAW264.7 macrophages, thereby inhibiting the occurrence of inflammation. These findings suggest that CVP‐I and CVP‐II hold potential applications in the fields of functional foods and pharmaceuticals.

## Graphical abstract

This study employed an ultrasonic‐assisted extraction (UAE) method combined with column chromatography purification to obtain two homogeneous polysaccharides (CVP‐I and CVP‐II) from *Conioselinum vaginatum*, which exhibited distinct physicochemical properties and structural characteristics, and exerted anti‐inflammatory effects by inhibiting the production of nitric oxide (NO), prostaglandin E2 (PGE_2_), interleukin‐6 (IL‐6), interleukin‐1β (IL‐1β), and tumor necrosis factor‐α (TNF‐α) in LPS‐induced RAW264.7 macrophages.

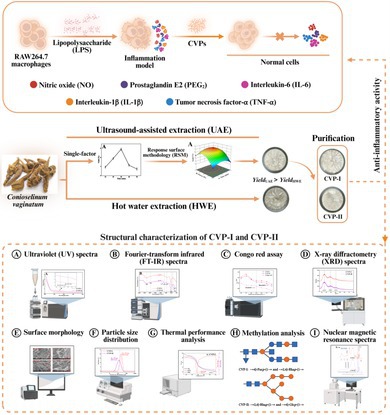

## 1 Introduction

Inflammation constitutes the fundamental defense mechanism by which the body combats pathogenic invasion and external injury, involving both innate and adaptive immune systems (Ma et al. 2020). It can be classified into two types, acute inflammation and chronic inflammation (Sanjeewa et al. 2021). Acute inflammation is characterized by neutrophil infiltration and the classic symptoms of redness, swelling, heat, and pain, whereas chronic inflammation is primarily marked by sustained macrophage activation, which is closely associated with the development of tissue fibrosis and neurodegenerative diseases (Padoan et al. 2023). Macrophages, as immune regulatory cells within the human body, play a defensive role in immune responses by modulating the dynamic equilibrium of pro‐inflammatory cytokines, chemokines, and inflammation‐related protein expression (Swanson et al. 2020). However, their excessive activation or dysfunction can lead to uncontrolled inflammatory responses, thereby triggering major diseases such as rheumatoid arthritis, type 2 diabetes and Alzheimer's disease (Gerasimova et al. 2022; Wang et al. 2025). Although traditional anti‐inflammatory drugs can alleviate symptoms, their prolonged use may trigger serious adverse reactions such as kidney damage, cardiovascular complications, and gastrointestinal harm (Phueanpinit et al. 2017). Therefore, the development of novel anti‐inflammatory therapies that combine high efficacy with safety has become an important direction in current research.

In recent years, natural compounds such as polysaccharides, polyphenols, and flavonoids have demonstrated significant advantages in the intervention of inflammatory diseases (Azab et al. 2016). Among these, polysaccharides demonstrate particularly outstanding performance in protecting cells from damage caused by inflammatory mediators, and are regarded as the most promising candidate substance with research potential for anti‐inflammatory applications (Kang et al. 2023). Polysaccharides, as macromolecular polymers widely present in nature, are composed of multiple identical or different monosaccharides linked by α‐ or β‐glycosidic bonds, and possess various biological activities such as antioxidant (Ji et al. 2020), anti‐inflammatory (Sun et al. 2020), antitumor (Ji, Peng, and Wang 2018), and anticancer (Wang and Yu 2022) effects, being extensively distributed throughout plants, animals and microorganisms (Li, Chen, et al. 2023).

*Conioselinum vaginatum* (*C. vaginatum*), a representative medicinal plant of the genus Ligusticum within the Apiaceae family, is primarily distributed across China, Kazakhstan, and Kyrgyzstan (Wei et al. 2022). In China, it is primarily distributed in the Yili and Tacheng regions of Xinjiang, accounting for over 50% of the global resources (Song et al. 2025). According to the Chinese Pharmacopeia, *C. vaginatum* has pharmacological effects such as anti‐inflammatory, analgesic, and antibacterial properties (Leong et al. 2020). In traditional applications, *C. vaginatum* is commonly administered as decoctions, medicinal wines, or powders to treat conditions such as inflammation, wind‐cold colds, headaches, and rheumatic arthralgia (Ma et al. 2019). However, despite the clear medicinal value of *C. vaginatum*, research on it remains relatively scarce at present, particularly concerning the structural analysis and biological activity of its polysaccharide components, which represent an unexplored area.

In the present study, *C. vaginatum* polysaccharides (CVP) were extracted from the rhizomes of *C. vaginatum* using an ultrasonic‐assisted extraction (UAE) method. On the basis of single‐factor experiments, the Box–Behnken design (BBD) in the response surface methodology (RSM) was employed to optimize the extraction process for CVP. Following separation and purification, two homogeneous polysaccharides (CVP‐I and CVP‐II) were obtained. The physicochemical properties and structural characteristics of CVP‐I and CVP‐II were systematically analyzed and compared by a comprehensive application of multiple techniques (HPGPC, GC, UV, FT‐IR, Congo red assay, XRD, SEM, particle size and zeta potential analysis, thermal stability analysis, methylation analysis, and NMR analysis). Furthermore, the anti‐inflammatory activity of the two polysaccharides was evaluated using an LPS‐induced inflammatory model in RAW264.7 macrophages. This study not only offers new insights for the efficient extraction, structural characterization, and anti‐inflammatory activity research of polysaccharides, but also provides a novel polysaccharide resource for the development of CVP components as immunomodulatory functional foods or therapeutic agents.

## 2 Materials and Methods

### 2.1 Materials and Reagents

The *C. vaginatum* specimen was purchased from a local market in the Yili region of Xinjiang, China, and identified by Professor Liu Wei of Yili Normal University. Currently preserved at the Key Laboratory of Yili Kazakh Medicinal Resource Exploration and Modernization Research (Lab No. WJL‐13).

The T‐series dextran (T‐10, T‐40, T‐70, T‐100, and T‐200 kDa) used in the experiment was purchased from Shanghai Biyuntian Biotechnology Co. Ltd. Standard monosaccharides (fucose, rhamnose, arabinose, xylose, mannose, glucose, and galactose) were purchased from Shanghai Lanjie Technology Development Co. Ltd. The mouse macrophage cell line RAW 264.7 was provided by the Cell Resource Centre of the Institute of Basic Medical Sciences, Chinese Academy of Medical Sciences. Lipopolysaccharide (LPS) from *Escherichia coli O111:B4* was purchased from Beijing Sol Biotech Co. Ltd. All other chemical reagents used were procured from China National Pharmaceutical Group Chemical Reagent Co. Ltd., and all reagents were of analytical grade.

### 2.2 Pretreatment

The dried rhizomes of *C. vaginatum* were ground and passed through a 40‐mesh sieve to obtain the powdered sample. It was then degreased for 24 h in petroleum ether (1:20, w/v) within a Soxhlet extractor, followed by two rounds of reflux extraction with 95% ethanol (24 h each) to remove small molecular substances. The filtered residue was air‐dried naturally and stored in a 4°C refrigerator for future use.

### 2.3 Extraction of CVP

#### 2.3.1 Ultrasound‐Assisted Extraction (UAE)

A 20 g sample of powder was extracted using an ultrasonic cell disruptor (JY92‐IIDN, Scientz, China) at an ultrasonic power of 200 W, a liquid–solid ratio of 40:1 mL/g, and a temperature of 50°C for 30 min. After extraction, the supernatant was collected. Under continuous stirring, trichloroacetic acid (TCA) was slowly added to reach a final concentration of 4% (w/v). The mixed solution was stirred continuously at 4°C for 20 min, then centrifuged (5000 rpm, 10 min), and the supernatant was collected again to remove proteins. Subsequently, the solution was concentrated to one‐third of its original volume, and threefold the volume of anhydrous ethanol was added to adjust its final concentration to 75% (v/v). The mixed solution was then placed at 5°C for 24 h to allow for precipitation. The precipitate was collected, followed by dialysis (MWCO 3500 Da) and freeze‐drying (−70°C, 48 h) to obtain the crude polysaccharides, named CVP. The yield was calculated according to Formula (1):

(1) $$\text{Yield}\left(\%\right)=\frac{C\times N\times V}{M}\times 100\%$$

where *C* is the polysaccharide concentration (mg/mL); *N* is the dilution factor; *V* is the extraction liquid volume (mL); *M* is the weight of raw materials (g).

#### 2.3.2 Single‐Factor Design

In this experiment, the ultrasonic power was first set to a constant value of 300 W. Using CVP yield as the evaluation metric, the effects of time (10, 20, 30, 40, and 50 min), liquid–solid ratio (10:1, 20:1, 30:1, 40:1, and 50:1 mL/g), and temperature (30°C, 40°C, 50°C, 60°C, and 70°C) on CVP yield were examined separately. After completing the first round of single‐factor experiments by selecting one variable at a time while holding all others constant, the optimal conditions were selected to proceed to the next round of single‐factor experiments until all single‐factor experiments were concluded.

#### 2.3.3 Box–Behnken Design (BBD)

Based on a single‐factor experimental design, the extraction process was further optimized using a BBD and response surface methodology (RSM) (Zhang et al. 2023). With the yield of CVP (Y) as the response value, time (A), ultrasonic power (B), liquid–solid ratio (C), and temperature (D) were selected as independent variables to establish a four‐factor, three‐level experimental design. The entire experimental design comprised 29 experimental points, including 24 analysis points and 5 replicate points, as shown in Table 1. Through multiple regression analysis, a second‐order polynomial equation was established to describe the relationship between the response value and the independent variables: *Y* = 9.17 – 0.03 *A* + 0.48 *B* – 0.20 *C* + 0.16 *D* + 0.02 *AB* – 0.03 *AC* + 0.03 *AD* + 0.03 *BC* + 0.03 *BD* + 0.01 *CD –* 0.65 *A* ^*2*^ – 1.14 *B* ^*2*^ – 0.62 *C* ^*2*^ – 0.21 *D* ^*2*^.

**TABLE 1:** BBD and the results for yield of CVP.

Run	A: time (min)	B: power (W)	C: liquid–solid ratio (mL/g)	D: temperature (°C)	Y: yield (%)
1	30	300	30:1	50	8.1
2	40	200	40:1	40	8.07
3	30	200	40:1	50	9.08
4	40	200	40:1	60	8.62
5	20	200	50:1	50	7.36
6	30	200	50:1	60	8.61
7	30	200	30:1	60	8.65
8	30	200	40:1	50	8.98
9	20	200	40:1	60	8.62
10	40	300	40:1	50	7.98
11	20	300	40:1	50	7.98
12	30	300	40:1	40	8.07
13	30	100	40:1	60	7.26
14	30	300	50:1	50	7.82
15	30	100	50:1	50	6.91
16	20	100	40:1	50	6.98
17	20	200	40:1	40	8.17
18	20	200	30:1	50	8.24
19	30	200	40:1	50	9.26
20	40	200	50:1	50	7.25
21	30	200	40:1	50	9.17
22	30	300	40:1	60	8.17
23	30	200	40:1	50	9.35
24	30	200	50:1	40	8.16
25	30	100	30:1	50	7.08
26	40	100	40:1	50	6.89
27	30	200	30:1	40	8.24
28	40	200	30:1	50	8.24
29	30	100	40:1	40	7.26

#### 2.3.4 Hot‐Water Extraction (HWE)

Under the optimized liquid–solid ratio of 38:1 mL/g established in the aforementioned experiment, extraction of the sample was performed using a magnetic stirrer (SP131325‐33Q, Thermo, USA) in a 100°C water bath for 80 min (HWE‐I). To establish a fair comparison, extraction of the sample was also performed using the same magnetic stirrer under the aforementioned experimentally optimized conditions of a time of 29 min, a liquid–solid ratio of 38:1 mL/g, and a temperature of 54°C (HWE‐II). All subsequent steps were consistent with those described in Section 2.3.1.

### 2.4 Isolation and Purification of CVP

CVP was dissolved in distilled water and subjected to preliminary purification using a DEAE‐650‐M cellulose column (52 × 360 mm). Gradient elution was performed using distilled water and NaCl solutions of varying concentrations (0.2, 0.4, 0.6 and 0.8 mol/L) as the mobile phase at a flow rate of 1 mL/min, with the major eluting components collected. Among these, the main peaks eluted with distilled water and 0.2 mol/L NaCl were designated as CVP‐I and CVP‐II, respectively. Further purification was carried out using a Sephadex G‐75 gel column (16 × 800 mm), with distilled water as the eluent at a flow rate of 0.4 mL/min. The homogeneous polysaccharide fraction was collected and pooled, then concentrated, dialyzed and freeze‐dried to obtain the purified polysaccharide.

### 2.5 Structural Characterization of CVP‐I and CVP‐II

#### 2.5.1 Components Determination

The total sugar content of polysaccharides was determined using the phenol‐sulfuric acid method (Yue et al. 2022). Briefly, 2 mg of the polysaccharide sample was mixed with 10 mL of a 5% phenol solution and 1 mL of concentrated sulfuric acid. After thorough mixing, the mixture was reacted in a 90°C water bath for 15 min, cooled to room temperature, and the absorbance value was measured at 490 nm. Total sugar content was calculated using the glucose standard curve (*Y* = 0.8893*X* − 0.0211, *R* ^*2*^ = 0.9962).

The protein content was determined using the Bradford method (Karimi et al. 2022). Bovine serum albumin (BSA) was used as the standard. A 1 mL aliquot of a 10 mg/mL polysaccharide solution was mixed with 5 mL of Coomassie Brilliant Blue solution. After shaking for 30 min, the absorbance value was measured at 595 nm. The protein content was calculated using the bovine serum albumin standard curve (*Y* = 0.7951 *X* + 0.0302, *R* ^*2*^ = 0.9813).

The uronic acid content was determined using the carbazole‐sulfuric acid method (Wang et al. 2022). A 2 mg polysaccharide sample was mixed with 1 mL of concentrated sulfuric acid and heated at 90°C for 30 min. After cooling, 100 μL of carbazole solution (0.1% w/v, prepared in ethanol) was added, and the mixture was reacted at room temperature for 80 min. The absorbance value was measured at 530 nm. The uronic acid content was calculated using the glucuronic acid standard curve (*Y* = 1.5426 *X* + 0.0738, *R* ^*2*^ = 0.9935).

#### 2.5.2 Molecular Weight Determination

The molecular weight (Mw) of CVP‐I and CVP‐II was determined using high‐performance gel permeation chromatography (HPGPC), with the specific methodology detailed in our research group's previously published literature (Cui et al. 2025). Analysis was performed using a Shimadzu LC‐400 HPLC system equipped with a TSK‐GEL G4000PWXL column (7.8 mm × 300 mm) and a RID‐10A refractive index detector. The chromatographic conditions were as follows: distilled water was used as the mobile phase at a flow rate of 0.6 mL/min, column temperature 35°C, injection volume 35 μL. The dextran standard was prepared using double‐distilled water at a concentration of 0.2%.

#### 2.5.3 Monosaccharide Composition Determination

The monosaccharide composition of CVP‐I and CVP‐II was determined based on the method described by Abuduwaili et al. (2022), with minor modifications. Specifically, 5 mg of each standard monosaccharide was accurately weighed, including fucose (Fuc), rhamnose (Rha), arabinose (Ara), xylose (Xyl), mannose (Man), glucose (Glc), and galactose (Gal). To each, 8 mg of hydroxylamine hydrochloride and 0.5 mL of pyridine were added, and the mixture was reacted in a 90°C water bath for 30 min. After cooling the solution to room temperature, 0.5 mL of acetic anhydride was added, and the reaction was continued in the 90°C water bath for another 30 min. Upon completion of the reaction, the supernatant was dried under a nitrogen stream, and the derivatives were dissolved in 1 mL of dichloromethane for gas chromatography (GC) (GC‐8890, Agilent, USA) analysis. The chromatographic conditions were as follows: detector temperature 270°C, injection port temperature 250°C; initial column temperature 160°C held for 5 min, then increased at 5°C/min to 190°C and held for 5 min, followed by a further increase at 3°C/min to 230°C and held for 5 min. For the CVP‐I and CVP‐II samples (5 mg each), 2 mL of 2 mol/L trifluoroacetic acid (TFA) was added, and hydrolysis was carried out at 100°C for 2 h (Wen et al. 2022; Xu et al. 2025). The hydrolyzed samples were then derivatized using the same procedure described above. Qualitative analysis was conducted by comparing the retention times with those of standard monosaccharides, and the molar percentages of each monosaccharide were calculated using the area normalization method (Jiang et al. 2025).

#### 2.5.4 UV and FT‐IR Spectra Determination

Aqueous solutions of CVP‐I and CVP‐II were prepared at a concentration of 1 mg/mL each, with distilled water as the blank. Their ultraviolet spectra were measured using a UV–visible spectrophotometer (UV2550, Shimadzu, Japan) within the wavelength range of 200–400 nm (Zhou et al. 2025).

CVP‐I and CVP‐II were separately mixed with KBr powder and ground to a particle size of approximately 1 mm. Spectral analysis was performed using a Fourier transform infrared spectrometer (NICOLET 6700, Thermo, USA) in the wavelength range of 4000–500 cm^−1^ (Wu et al. 2022).

#### 2.5.5 Congo Red Determination

The Congo red assay was performed according to the method of Fan et al. (2023) with minor modifications. Briefly, 1 mL of 3.0 mg/mL CVP‐I and CVP‐II aqueous solutions were each mixed with 1 mL of 2.0 mol/L Congo red solution. After reacting in the dark for 30 min, 1 mL of NaOH solutions at different concentrations (0, 0.1, 0.2, 0.3, 0.4, 0.5, and 0.6 mol/L) was added to each mixture. Distilled water was used as the control group. Subsequently, the maximum absorption wavelength (λ_max_) within the 400–600 nm range was recorded using a UV–visible spectrophotometer.

#### 2.5.6 XRD Determination

The dried CVP‐I and CVP‐II (30 mg each) were uniformly ground, then separately filled into sample chambers (20 mm × 18 mm) and compacted using glass presses to render the surface flat and dense. Subsequently, the crystal structures of CVP‐I and CVP‐II were analyzed using an X‐ray powder diffractometer (D8 Advance, Bruker, Germany) (Ding et al. 2025). The analysis conditions were as follows: a scanning range 2θ of 5°–80°, a step size of 0.01°, and a scanning speed of 0.1 s/step.

#### 2.5.7 Morphology Characteristics Determination

In brief, 1 mg of dried CVP‐I and CVP‐II were separately mounted on the sample stage with conductive adhesive. After being coated with a platinum conductive film via ion sputtering deposition, the samples were examined using a scanning electron microscope (SEM) (7500 F, Joel, Japan) under conditions of an acceleration voltage of 10 kV and a working distance of 8 mm, with image magnifications of 500× and 5000× (Al‐Wraikat et al. 2022).

#### 2.5.8 Particle Size and Zeta Potential Determination

CVP‐I and CVP‐II were prepared as aqueous solutions with a concentration of 1.0 mg/mL each, and then the particle size and zeta potential of each sample were measured using a Zetasizer Nano ZS laser nanoparticle size analyzer (Malvern, Worcestershire, UK) at a temperature of 25°C and an equilibration time of 2 min (Bu et al. 2024).

#### 2.5.9 Thermal Performance Determination

Thermal performance analysis of CVP‐I and CVP‐II was conducted using a simultaneous thermal analyzer (STA200, Joel, Japan), including thermogravimetric analysis (TG), differential thermogravimetric analysis (DTG), and differential scanning calorimetry (DSC) (Abuduwaili et al. 2019). During the experiment, exactly 5 mg of the polysaccharide sample was weighed into a platinum crucible. Under a nitrogen atmosphere, the temperature was programmed to increase from 25°C to 600°C at a rate of 10°C/min.

#### 2.5.10 Methylation Determination

Methylation analysis of CVP‐I and CVP‐II was performed according to the method reported by Ji et al. (2020) with minor modifications. Specifically, 10 mg each of dried CVP‐I and CVP‐II were dissolved separately in 2 mL of distilled water, and the solutions were adjusted to a pH of 4–4.5 using 0.1 mol/L hydrochloric acid (HCl). 20 mg of carbodiimide (EDC) was added, and the reaction was maintained within this pH range for 1 h. Subsequently, 30 mg of deuterated sodium borohydride (NaBD₄) was added, and the reaction was continued at room temperature for 3 h. Following conclusion of the reaction, the reaction solution was neutralized with 25% acetic acid, then dialyzed and freeze‐dried to obtain the pre‐reduced polysaccharide samples. Separately, 5 mg of pre‐reduced CVP‐I and CVP‐II were separately and completely dissolved in 2 mL of anhydrous dimethyl sulfoxide, followed by the addition of 40 mg of NaOH powder. The reaction was carried out under nitrogen protection for 4 h. Subsequently, 1 mL of iodomethane was slowly added in an ice‐water bath, and the reaction continued for another 3 h under dark conditions. After the reaction, the mixture was extracted with 2 mL of dichloromethane. The extract was dehydrated over a sodium sulfate column (0.5 cm × 15 cm) and then hydrolyzed at 100°C for 6 h. Then, 0.5 mL of NaBD₄ solution (1 mol/L) was added and reduction was performed for 3 h. After neutralization with 25% acetic acid, 2.5 mL of acetic anhydride was added and thoroughly mixed, and acetylation reaction was conducted at 100°C for 2.5 h. Finally, the mixture was extracted five times with dichloromethane, and the organic phase was collected into a GC vial. Analysis was conducted using a GC–MS (7820A, Agilent, USA) equipped with an HP‐5 MS column (30 m × 250 mm × 0.25 μm). The GC–MS conditions were initial temperature 120°C, and then increased at 2°C/min to 210°C, and held for 2 min. The types of sugar residues were identified by comparing the mass spectrometry results with the CCRC PMAA spectral database, and the molar percentages of each component were calculated based on the corresponding GC peak areas.

#### 2.5.11 NMR Spectra Determination

In this experiment, 30 mg each of CVP‐I and CVP‐II were weighed and dissolved separately in 1.0 mL of deuterated water (D_2_O, 99.9%). The prepared solutions were transferred to NMR tubes for analysis. Subsequently, ^1^H NMR (600 MHz) and ^13^C NMR (150 MHz) spectra were acquired at 25°C using an NMR spectrometer (Bruker AVANCE AV‐600, Rheinstetten, Germany) equipped with an ultra‐low temperature probe (Liu et al. 2024). DSS (2,2‐dimethyl‐2‐silapentane‐5‐sulfonate) was used as the internal standard, with chemical shifts expressed in ppm.

### 2.6 Anti‐Inflammatory Activity Determination

#### 2.6.1 Culture of RAW264.7 Macrophages and Establishment of an Inflammation Model

The RAW 264.7 cell line was cultured in RPMI‐1640 medium supplemented with 10% fetal bovine serum (FBS) and antibiotics (penicillin 100 U/mL, streptomycin 100 μg/mL) at 37°C in a humidified incubator with 5% CO_2_. The culture medium was replaced every 2 days.

The establishment of the RAW264.7 macrophages inflammatory model was based on the method described by Wei et al. (2021), with minor modifications. Briefly, cells in the logarithmic growth phase were seeded at a density of 1 × 10^4^ cells/mL (100 μL per well) in a 96‐well plate. After overnight incubation, the medium was replaced with serum‐free RPMI‐1640 medium and incubated for 2 h. Subsequently, 2 μg/mL LPS was added to induce an inflammatory response for 24 h, thereby establishing an inflammatory model in RAW264.7 macrophages. To validate this model, dexamethasone (1 μM) was employed as a positive control in the experiment.

#### 2.6.2 RAW264.7 Macrophages Viability Determination

The MTT assay was employed to assess the viability of RAW264.7 macrophages, following the methodology described by Chen et al. (2023) with minor modifications. Specifically, RAW264.7 macrophages cultured as described in Section 2.6.1 and divided into four groups: the sample group (RAW264.7 macrophages co‐treated with 2 μg/mL LPS and 10, 50, 100, 200, or 400 μg/mL of either CVP‐I or CVP‐II), the blank group (untreated RAW264.7 macrophages), the control group (RAW264.7 macrophages treated with 2 μg/mL LPS), and positive control group (RAW264.7 macrophages co‐treated with 2 μg/mL LPS and 1 μM dexamethasone). After centrifugation and removal of the culture medium, 20 μL of 5 mg/mL MTT solution and 180 μL of fresh medium were added to each well. Following 4 h of incubation, the supernatant was removed, and 150 μL of DMSO was added to dissolve the formazan crystals. The absorbance was measured at 490 nm using a microplate reader (MR‐96A, Mindray, China). Cell viability was calculated according to formula (2):

(2) $$\text{Cell viability}\left(\%\right)=\frac{A_{s}-A_{b}}{A_{c}-A_{b}}\times 100\%$$

where *A*_*s*_, *A*_*b*_, *A*_*c*_ and are the absorbance values of the sample group, blank group, and control group, respectively.

#### 2.6.3 NO, PGE_2_ and Pro‐Inflammatory Cytokines Determination

RAW264.7 macrophages culture and grouping methods were performed as described in Sections 2.6.1 and 2.6.2. The levels of NO, PGE_2_, and pro‐inflammatory cytokines in the culture medium supernatant were measured using the nitrate reductase method (NO detection kit) and the ELISA method (PGE_2_, IL‐6, IL‐1β, and TNF‐α detection kits), following the manufacturers' instructions for all procedures (Ma et al. 2020; Sun et al. 2020).

### 2.7 Statistical Analysis

All experiments were independently repeated three times (*n* = 3). Data are expressed as mean ± standard deviation (SD). All images without error bars are representative images from three experiments. Analysis of variance (ANOVA) was performed using Duncan's multiple range test, and significance levels (*p* < 0.05) were indicated by different letters (A, a, and b) and symbols (* and #). Anti‐inflammatory activity images were created using GraphPad Prism 10.0 software, NMR spectra data were processed using MestReNova software, and all other curves were plotted using Origin 2025 software.

## 3 Results and Discussion

### 3.1 Impact of Different Factors on UAE

**FIGURE 1:**
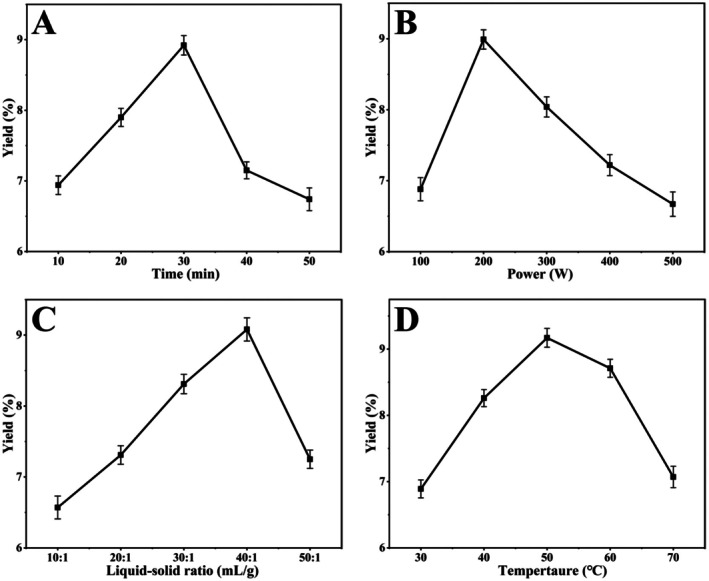
Effect of time (A), ultrasonic power (B), liquid–solid ratio (C), and temperature (D) on CVP yield.

The extraction process of polysaccharides is time‐dependent. Insufficient extraction time results in incomplete dissolution of polysaccharides. With the extension of time, the sustained ultrasonic cavitation effect and microbubble rupture intensify the disruption of plant cell walls, reducing the constraints imposed by cellular structures on mass transfer processes, thereby promoting the diffusion and release of polysaccharides into the solvent (Li et al. 2024). However, excessively prolonged extraction times not only increase energy consumption but may also cause partial degradation of the dissolved polysaccharides due to thermal effects or excessive cavitation, whilst simultaneously accelerating the dissolution of impurities, such as small‐molecule pigments and soluble proteins, ultimately reducing polysaccharide yield (Yu et al. 2023). As shown in Figure 1A, the CVP yield reached a maximum of 8.92% ± 0.24% at an extraction time of 30 min, after which it exhibited a marked downward trend. Therefore, a time range of 20–40 min was selected for subsequent optimization.

Ultrasonic power directly influences polysaccharide yield. Within the appropriate power range, compared to lower power, increasing ultrasonic power promotes the generation of more cavitation bubbles, thereby elevating the pressure throughout the extraction system and further facilitating cell wall rupture and the release of polysaccharides (Yang et al. 2024). Conversely, if the power exceeds this range, excessively high power may generate an excessive number of free radicals, potentially triggering side reactions that lead to polysaccharide degradation, thereby resulting in a decrease in polysaccharide yield (Kang et al. 2023). As shown in Figure 1B, the CVP yield increased significantly with rising ultrasonic power, reaching a peak of 8.99% ± 0.21% at 200 W, after which it gradually decreased. Therefore, an ultrasonic power range of 100–300 W was selected for subsequent optimization.

Polysaccharides possess large molecular weights and tend to aggregate, thus requiring a sufficient amount of solvent for effective dissolution. Within the appropriate liquid–solid ratio range, increasing the liquid–solid ratio reduces the solution's concentration and viscosity, thereby elevating the osmotic pressure across the cell membrane, enhancing the driving force for polysaccharide mass transfer, and accelerating polysaccharide dissolution (Jiang et al. 2024). Concurrently, the increased solvent volume expands the effective area of ultrasonication, thereby enhancing cavitation efficiency (Yu, Li, et al. 2024). However, an excessively high liquid–solid ratio dilutes the solution and weakens intermolecular interactions, thereby reducing the thermal transfer and osmotic effects within the ultrasonic system, ultimately leading to a decrease in polysaccharide yield (Jiang et al. 2025). As shown in Figure 1C, the CVP yield increases with the rising liquid–solid ratio, reaching a maximum of 9.08% ± 0.28% at 40:1 mL/g. Beyond this ratio, the yield gradually decreases. Therefore, a liquid–solid ratio range of 30:1–50:1 mL/g was selected for subsequent optimization.

Temperature is a key factor influencing polysaccharide yield. Moderate temperature elevation helps reduce solvent viscosity and surface tension, while enhancing the diffusion rate of polysaccharide molecules, thereby lowering the intensity threshold required for ultrasonic cavitation effects, subsequently promoting the disruption of cell wall structures to facilitate polysaccharide dissolution and extraction (Liu et al. 2017). However, excessively high temperatures may induce thermal effects, which can cause the glycosidic bonds of polysaccharides to break and undergo thermal degradation (Yu, Li, et al. 2024). Moreover, high temperatures also significantly increase the solubility of impurities, such as proteins, phenolic compounds, and starch, which not only affects polysaccharide purity but also leads to a reduction in polysaccharide yield (Song et al. 2025). As shown in Figure 1D, the CVP yield exhibits an initial increase followed by a decrease with rising temperature, reaching a maximum of 9.17% ± 0.22% at 50°C. Therefore, a temperature range of 40°C–60°C was selected for subsequent optimization.

### 3.2 Optimization of the UAE Process

Table 2 presents the ANOVA results for the model. The extremely significant *p*‐value (*p* < 0.0001), the high *F*‐value (*F* = 16.30), and the not significant lack of fit term (*F* = 3.68 > 0.05; *p* = 0.1106 > 0.05) indicate that the experimental results align well with the predicted values of the polynomial model and are statistically significant (Li et al. 2024). Additionally, the proximity of *R* ^*2*^ = 0.9422 and *R* ^2^_adj_ = 0.8844 confirms the accuracy and general applicability of the model (Zhang et al. 2023). The low coefficient of variation (*CV* = 3.07%) further demonstrates that the experimental results of this model exhibit good reliability and high precision (Yang et al. 2024). The order of influence of each factor on polysaccharide yield was: ultrasonic power (B) > liquid–solid ratio (C) > temperature (D) > time (A), with factor B exhibiting the most significant impact (*p* < 0.01).

**TABLE 2:** ANOVA for response surface quadratic model for the yield of CVP.

Source	Sum of squares	Df	Mean square	*F*‐value	*p*‐Value	Significance
Model	14.06	14	1	16.30	< 0.0001	**
*A*	0.0075	1	0.0075	0.1217	0.7324	
*B*	2.75	1	2.75	44.55	< 0.0001	**
*C*	0.4961	1	0.4961	8.05	0.0132	*
*D*	0.3201	1	0.3201	5.19	0.0389	*
*AB*	0.002	1	0.002	0.0329	0.8588	
*AC*	0.003	1	0.003	0.0491	0.8279	
*AD*	0.0025	1	0.0025	0.0406	0.0433	*
*BC*	0.003	1	0.003	0.0491	0.0379	*
*BD*	0.0025	1	0.0025	0.0406	0.0117	*
*CD*	0.0004	1	0.0004	0.0065	0.0369	*
*A* ^*2*^	2.72	1	2.72	44.16	< 0.0001	**
*B* ^*2*^	8.36	1	8.36	135.64	< 0.0001	**
*C* ^*2*^	2.46	1	2.46	39.84	< 0.0001	**
*D* ^*2*^	0.2867	1	0.2867	4.65	0.0489	*
Residual	0.8628	14	0.0616			
Lack of fit	0.7782	10	0.0778	3.68	0.1106	
Pure error	0.0847	4	0.0212			
Cor total	14.92	28				

*Note: R* ^2^ = 0.9422; *R* ^2^_adj_ = 0.8844; *C.V*.% = 3.07.

* *p* < 0.05.

** *p* < 0.01.

**FIGURE 2:**
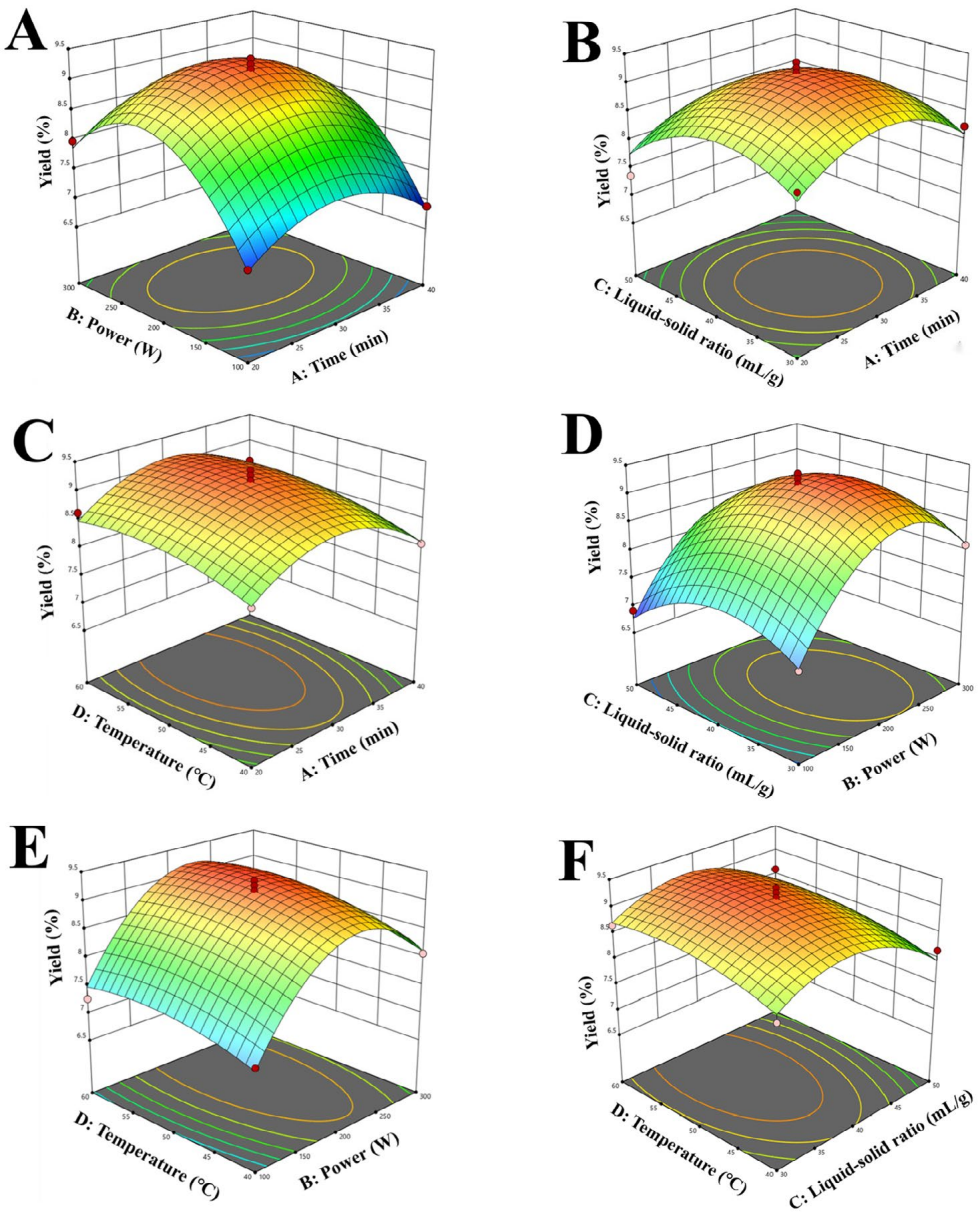
Response surface plots of interactions between different factors (A–F).

Generally, the steeper gradient on a three‐dimensional response surface indicates a more significant influence of the factor, while the more elliptical the shape of a two‐dimensional contour line reflects a stronger interaction between the corresponding variables (Cui et al. 2018; Kang et al. 2023). Based on the steepness of the response surface plots (Figure 2), among the four variables, B exerts the most significant influence on polysaccharide yield, followed by C, D, and A. Analysis of the contour plots (Figure S1) shows that the contour shapes between AD, BC, BD, and CD exhibit distinct elliptical form with dense distribution, indicating statistically significant interactive effects. In contrast, the contour lines for the AB and AC interactions are nearly circular, suggesting their effects are not significant. These findings are consistent with the ANOVA results presented in Table 2.

### 3.3 Model Validation and Comparison of Different Extraction Methods

The optimal extraction parameters predicted by Design Expert 13.0 software were: time 28.83 min, ultrasonic power 204.45 W, liquid–solid ratio 38.12:1 mL/g, and temperature 54.24°C, with a predicted CVP yield of 10.23%. Considering the feasibility of experimental operations, the parameters were adjusted to a time of 29 min, ultrasonic power of 204 W, liquid–solid ratio of 38:1 mL/g, and temperature of 54°C. Under these conditions, the average CVP yield obtained from validation experiments was 10.12% ± 0.38% (*n* = 3), which closely matches the predicted value. This indicates that the model possesses good predictive accuracy for the extraction process.

Table 3 summarizes the comparative results of CVP obtained from UAE and HWE in terms of yield, total sugar content, protein content, and uronic acid content. Under the same time, liquid–solid ratio, and temperature conditions, the CVP yield of UAE (10.12% ± 0.38%) was significantly higher than that of HWE‐II (3.31% ± 0.08%) (*p* < 0.05). This result is consistent with the conclusion reported by Li et al. (2024) in their study of *Angelica dahurica* Radix polysaccharides, where UAE (8.71%) was found to be superior to HWE (6.57%). Notably, although the yield obtained by HWE‐I (6.48% ± 0.26%) was significantly higher than that of HWE‐II, it remained markedly lower than that of UAE (*p* < 0.05).

**TABLE 3:** Effects of different extraction methods on yield and composition of CVP.

Extraction methods	UAE	HWE	
		HWE‐I	HWE‐II
Time (min)	29	80	29
Power (W)	204	/	/
Liquid–solid ratio (mL/g)	38:1	38:1	38:1
Temperature (°C)	54	100	54
Yield (%)	10.12 ± 0.38^a^	6.48 ± 0.26^b^	3.31 ± 0.08^c^
Total sugar (%)	56.14 ± 0.91^a^	41.34 ± 0.78^b^	48.67 ± 0.82^c^
Protein (%)	2.13 ± 0.04^a^	1.83 ± 0.02^a^	2.01 ± 0.04^a^
Uronic acid (%)	6.24 ± 0.08^a^	3.11 ± 0.06^b^	5.23 ± 0.09^a^

*Note:* “/” indicates non‐existence. Data are expressed as mean ± SD (*n* = 3). Values without the same superscript letters indicate significant differences (*p* < 0.05).

Moreover, the total sugar content (56.14% ± 0.91%), protein content (2.13% ± 0.04%), and uronic acid content (6.24% ± 0.08%) of the CVP obtained by UAE were all higher than those obtained by HWE‐II (48.67% ± 0.82%, 2.01% ± 0.04%, and 5.23% ± 0.09%, respectively). This may stem from the cavitation bubbles generated by ultrasound, which facilitate the dissolution of active components such as polysaccharides (Wei and Zhang 2023). Similarly, the total sugar, protein, and uronic acid content obtained by HWE‐II were all higher than those of HWE‐I (41.34% ± 0.78%, 1.83% ± 0.02%, and 3.11% ± 0.06%, respectively). These data demonstrate that UAE and HWE‐II, by using an appropriate extraction time (29 min) and temperature (54°C), help reduce degradation of active components, thereby better preserving the structural integrity of each constituent, with UAE being the most effective. In contrast, the longer extraction time (80 min) and high temperature treatment (100°C) in HWE‐I may lead to polysaccharide depolymerization, protein denaturation, and degradation of uronic acid, thereby reducing the content of target components (Li, Zhang, et al. 2023). The results indicate that compared with HWE, UAE not only significantly enhances polysaccharide yield but also effectively preserves the bioactive components and structural integrity of polysaccharides. Therefore, UAE is a more advantageous CVP extraction process.

### 3.4 Isolation and Purification of CVP

**FIGURE 3:**
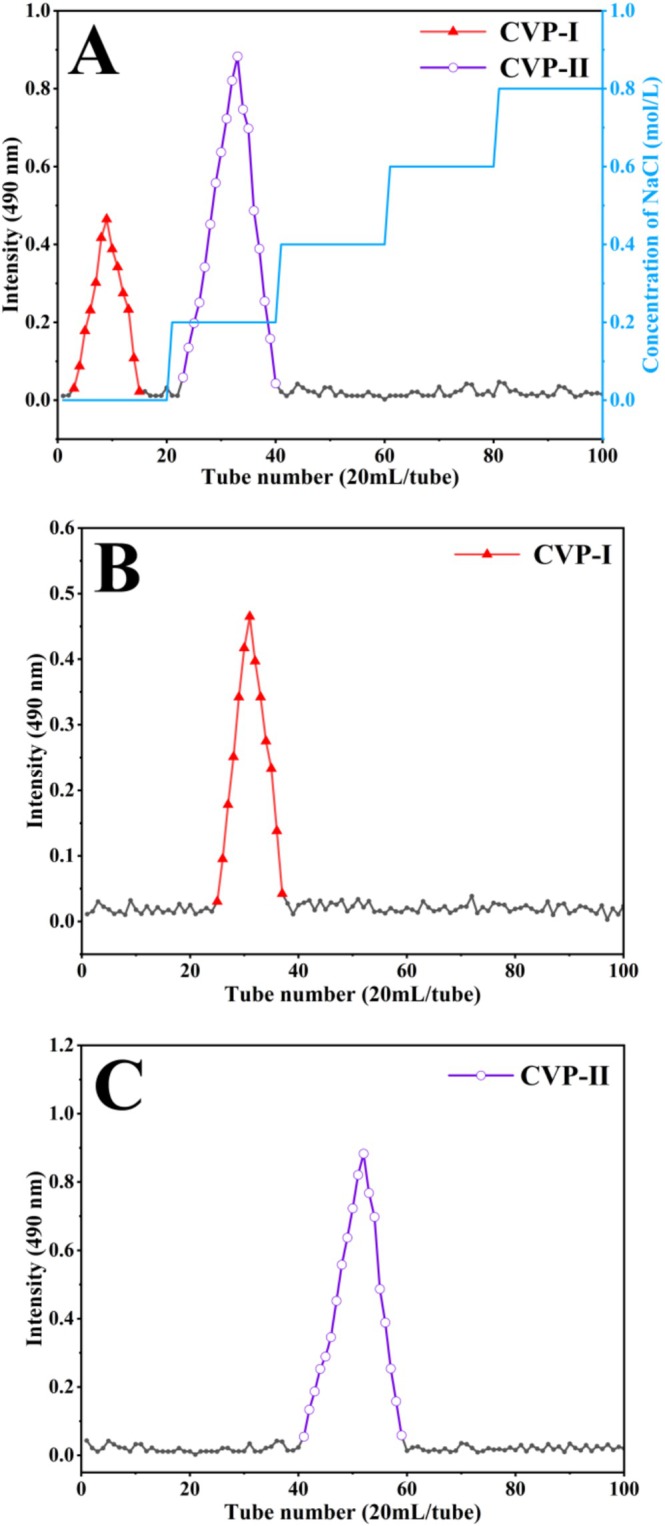
Purification elution curve of CVP. DEAE‐650‐M cellulose column elution curve (A), Sephadex G‐75 gel column elution curves (B, C).

Figure 3A shows the distilled water eluate fraction (named CVP‐I, yield 26.11% ± 0.27%) and the 0.2 mol/L NaCl eluate fraction (named CVP‐II, yield 58.74% ± 0.51%) obtained by purifying CVP through a DEAE‐650‐M cellulose column. After collecting the two fractions, further purification was carried out by Sephadex G‐75 column chromatography, yielding two distinct elution peaks (Figure 3B,C). Following concentration, dialysis, and freeze‐drying, homogeneous CVP‐I and CVP‐II were ultimately obtained with yields of 22.37% ± 0.39% and 41.53% ± 0.62%, respectively.

### 3.5 Structural Analysis of CVP‐I and CVP‐II

#### 3.5.1 Chemical Composition Analysis

Table 4 summarizes the chemical composition of CVP‐I and CVP‐II, including total sugar content, protein content, uronic acid content, Mw, and monosaccharide composition.

**TABLE 4:** Chemical composition analysis of CVP‐I and CVP‐II.

Sample	Total sugar (%)	Protein (%)	Uronic acid (%)	Mw (kDa)	Monosaccharide composition (mol‐%)						
					Fuc	Rha	Ara	Xyl	Man	Glc	Gal
CVP‐I	78.27 ± 0.41^a^	/	/	26.42 ± 0.37^a^	42.17	27.52	17.13	/	4.87	8.31	/
CVP‐II	91.79 ± 0.58^b^	/	0.76 ± 0.02	17.81 ± 0.23^b^	19.68	33.17	16.74	/	8.04	22.37	/

*Note:* “/” indicates not detected. Data are expressed as mean ± SD (*n* = 3). Values without the same superscript letters indicate significant differences (*p* < 0.05).

The analysis results showed that the total sugar content of CVP‐II (91.79% ± 0.58%) was significantly higher than that of CVP‐I (78.27% ± 0.41%) (*p* < 0.05), which is consistent with the findings reported by Wang et al. (2023) in their study on polysaccharides from *Choerospondias axillaris* peels, where the salt‐eluted fraction polysaccharides exhibited higher total sugar content. Notably, no protein and uronic acid were detected in CVP‐I, while CVP‐II contained only trace amounts of uronic acid (0.76% ± 0.02%) and no protein residues, indicating high purity for both polysaccharides.

Mw determination showed that the Mw of CVP‐II was 17.81 ± 0.23 kDa, significantly lower than that of CVP‐I (26.42 ± 0.37 kDa) (*p* < 0.05). This may stem from the disruption of hydrogen bonds and electrostatic interactions between polysaccharide molecular chains during the NaCl elution process, leading to the release of low Mw components. Similarly, Rostami and Gharibzahedi (2016) reported in their study of jujube polysaccharides that JCP‐2 (NaCl elution fraction, 9.1 × 10^4^ Da) exhibited a lower Mw than JCP‐1 (deionized water elution fraction, 1.5 × 10^5^ Da). Additionally, the purification elution curves (Figure 3B,C) and Mw distribution curves (Figure S2) for both polysaccharides exhibited clear and symmetrical profiles, confirming their relatively homogeneous Mw distribution (Zhou et al. 2020).

**FIGURE 4:**
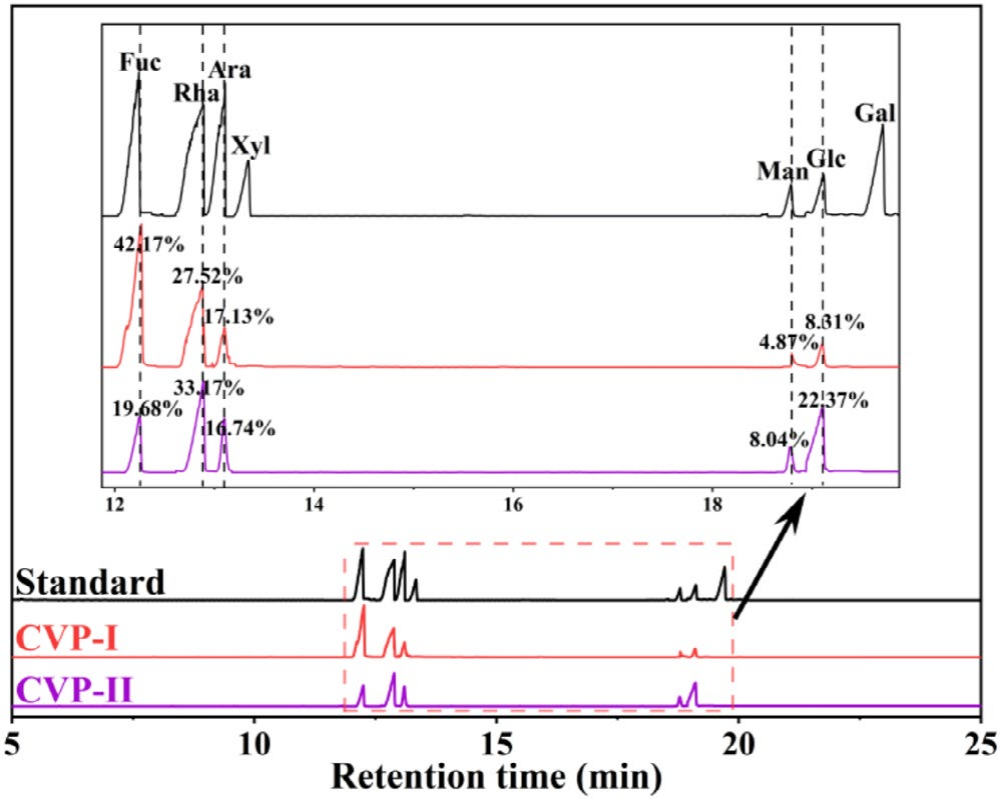
Monosaccharide composition of CVP‐I and CVP‐II from GC.

Monosaccharide composition analysis results (Table 4 and Figure 4) showed that both CVP‐I and CVP‐II were composed of five monosaccharides, including Fuc, Rha, Ara, Man, and Glc, but there were significant differences in their monosaccharide percentages (*p* < 0.05). Specifically, compared to CVP‐I, CVP‐II exhibited a significant decrease in Fuc content (−22.49%) and a substantial increase in Glc content (14.06%), while the levels of other monosaccharides remained relatively unchanged. This phenomenon may be related to the selective disruption of Fuc structures in the polysaccharides during NaCl elution process, while preserving more Glc‐containing component (Chen et al. 2020). This is consistent with the findings reported by Song et al. (2025) in their study on pumpkin polysaccharides. Furthermore, the molar ratio of monosaccharides in CVP‐I was 8.76:5.65:3.52:1:1.71, whereas those in CVP‐II was 2.45:4.13:2.08:1:2.78. Notably, differences in the monosaccharide composition may influence the biological activity of polysaccharides. Research indicates that Fuc and Glc are positively correlated with anti‐inflammatory activity (Wang et al. 2024). Among these, polysaccharides rich in Fuc may rely more heavily on modulating Toll‐like receptor 4 (TLR4)‐mediated signaling pathways, thereby influencing the polarization state of macrophages and consequently regulating the inflammatory response process (Chang et al. 2023). In contrast, polysaccharides with high Glc content may exert their anti‐inflammatory effects primarily by activating macrophages, modulating signaling pathways such as nuclear factor κB (NF‐κB)/mitogen‐activated protein kinase (MAPK), and promoting the production of anti‐inflammatory cytokines (Chen et al. 2023). Similarly, the content of Rha, Ara, and Man also influences anti‐inflammatory activity (Qiu et al. 2025). Therefore, the differences in monosaccharide composition between CVP‐I and CVP‐II may lead to the variation in their anti‐inflammatory activities.

#### 3.5.2 UV and FT‐IR Spectra Analysis

**FIGURE 5:**
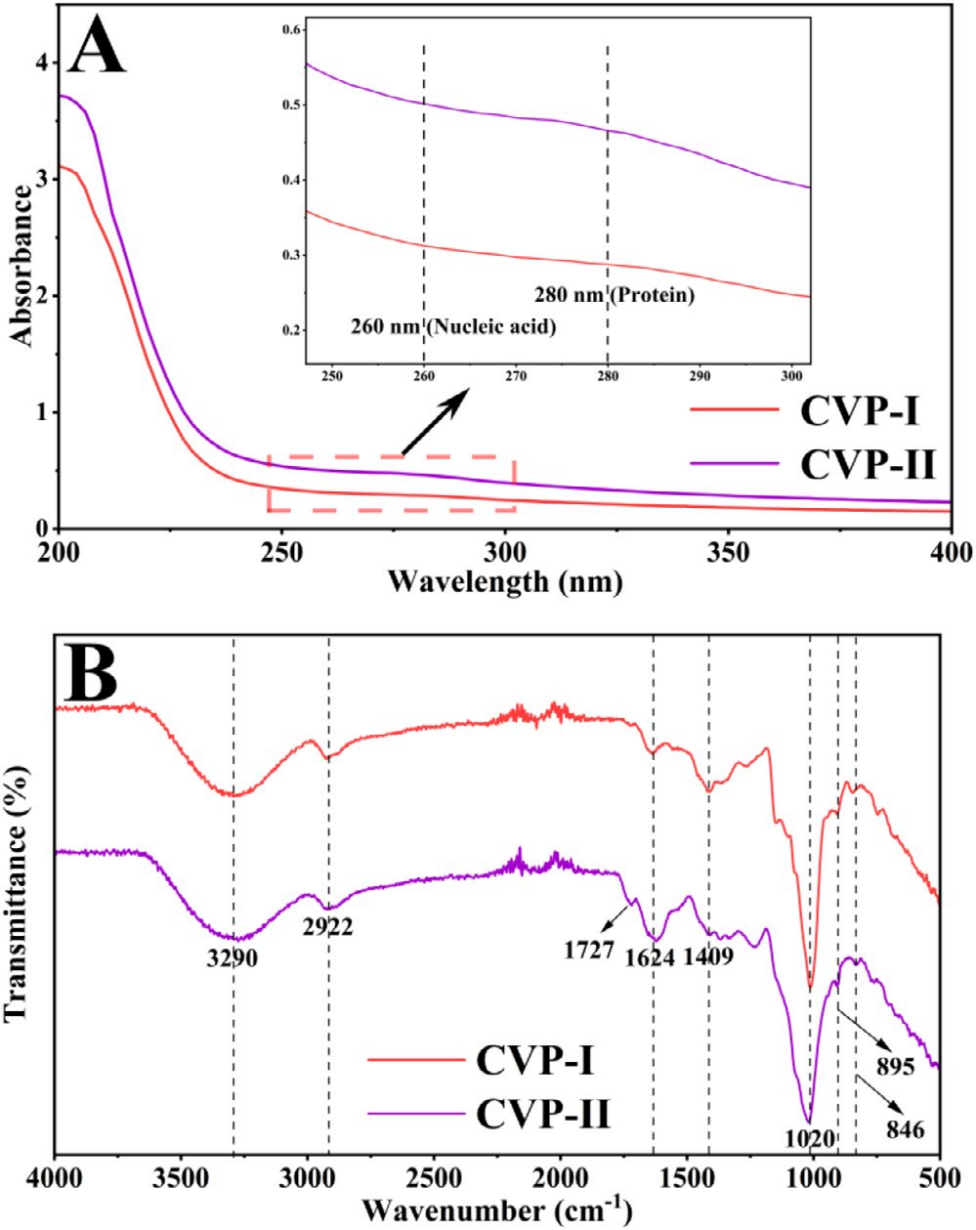
UV spectra (A) and FT‐IR spectra (B) of CVP‐I and CVP‐II.

UV spectra serve as an initial screening tool for polysaccharide structural research, effectively detecting the presence of impurities such as nucleic acids, proteins, and phenolic compounds. As shown in Figure 5A, the UV spectra of both CVP‐I and CVP‐II revealed no absorption peaks at 260 nm, indicating the absence of nucleic acid components (Wei et al. 2021). In addition, no discernible absorption peaks were detected at 280 nm for both CVP‐I and CVP‐II, suggesting the presence of no residual protein components (Chen et al. 2023). This finding is consistent with the results of the protein content analysis in Table 4.

The identification of polysaccharides typically commences with their distinctive functional groups or structures, for which FT‐IR spectroscopy is an effective means. As shown in Figure 5B, both CVP‐I and CVP‐II exhibit similar absorption peaks in the range of 4000–500 cm^−1^, indicating that they share common structural features. Among them, the broad and strong absorption peak at 3290 cm^−1^ is attributed to the O‐H stretching vibration of the hydroxyl group, while the weak absorption peak at 2922 cm^−1^ arises from the asymmetric stretching vibration of C‐H bonds (Liu et al. 2022). The absorption near 1624 cm^−1^ may originate from the O‐H bending vibration of bound water or the amide I band of proteins (Fan et al. 2023). The peak at 1409 cm^−1^ corresponds to the asymmetric stretching vibration of the COO^−^ group, while the absorption peak near 1020 cm^−1^ originates from the asymmetric stretching vibrations of C‐O‐H and C‐O‐C, confirming the presence of pyranose ring structures in both polysaccharides (Li, Chen, et al. 2023). Moreover, characteristic peaks at 895 and 846 cm^−1^ further confirm the presence of both α‐ and β‐glycosidic bonds in these two polysaccharides (Lin et al. 2023).

Although the two polysaccharides share similarities, they also exhibit significant differences. Compared to CVP‐I, CVP‐II shows a weak absorption peak at 1727 cm^−1^, which may be attributed to the stretching vibration of the carbonyl group (C=O), indicating the presence of trace amounts of uronic acid components (Xiong et al. 2023). This result is mutually corroborated with the findings from chemical composition analysis and UV spectra analysis. Notably, CVP‐I and CVP‐II exhibit stronger absorption intensities at 1409 and 1624 cm^−1^, respectively. These results indicate that the two polysaccharides share common structural features while each exhibits distinct structural characteristics.

#### 3.5.3 Congo Red Analysis

**FIGURE 6:**
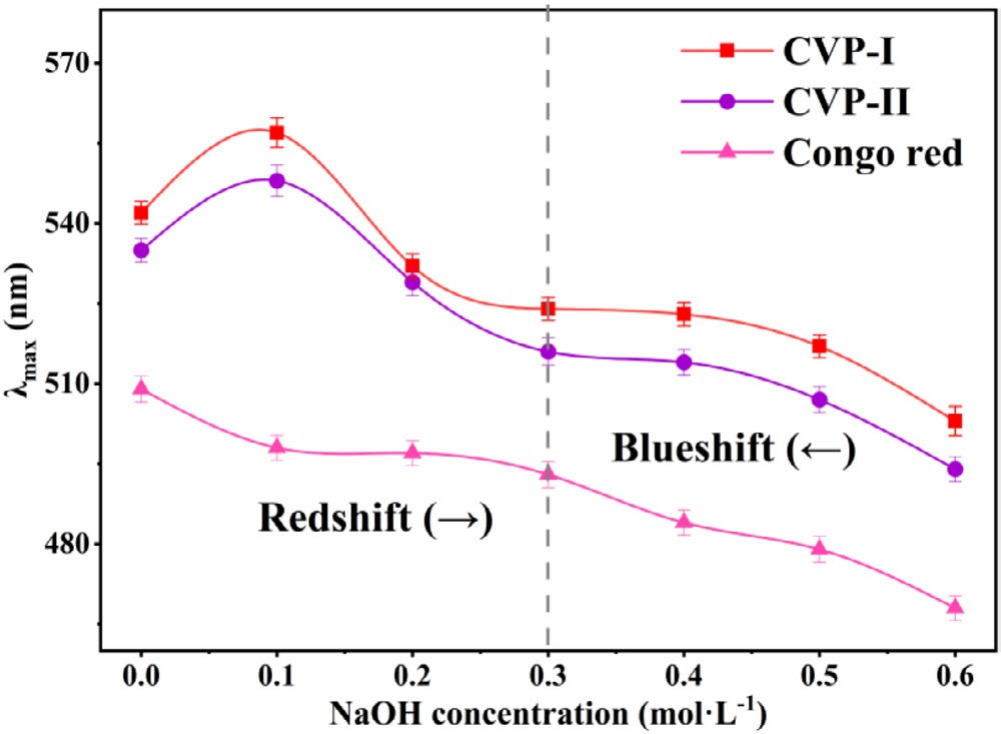
Variation in the λ_max_ of Congo red‐polysaccharide complexes in NaOH solutions of different concentrations.

The Congo red assay is a reliable method for detecting the triple‐helix structure of polysaccharides. In a low concentration of NaOH solution (0.1–0.3 mol/L), polysaccharides with an ordered helical conformation form complexes with Congo red, resulting in a significant red shift of the λ_max_ (Jiang et al. 2025). As the concentration of NaOH solution continues to increase (> 0.3 mol/L), the disruption of intra‐ and inter‐molecular hydrogen bonds in polysaccharides induces a conformational shift from triple helix to single‐chain random coil, thereby causing a synchronous blue shift in λ_max_ (Abuduwaili et al. 2022). As shown in Figure 6, both CVP‐I and CVP‐II exhibit the characteristic red shift followed by a blue shift within the NaOH concentration range of 0.1–0.6 mol/L, which is in sharp contrast to the control group (Congo Red solution mixed with distilled water). This result indicates that both polysaccharides possess a stable triple‐helix structure. This structure stabilizes the conformations of CVP‐I and CVP‐II through interchain hydrogen bonds and hydrophobic interactions, endowing them with excellent thermal stability and resistance to enzymatic degradation (Meng et al. 2020). This enables both polysaccharides to withstand high temperatures, extreme pH levels, and enzymatic environments during food processing and digestion, making them suitable for the development of functional beverages, baked goods, and enteral nutrition formulations (Meng et al. 2020). Furthermore, the triple‐helix structure of polysaccharides is closely associated with their anti‐inflammatory activity. Du et al. (2017) reported that oral administration of *Schizophyllum commune* polysaccharides with a triple‐helix conformation significantly alleviated dextran sulfate sodium (DSS)‐induced colitis in mice and reduced macrophage infiltration in inflamed tissues. Similarly, Chen et al. (2025) isolated FVPU1 and FVPU2 from *Flammulina velutipes* polysaccharides. Among them, FVPU2, with a more stable triple‐helix structure, demonstrated superior efficacy compared to FVPU1 in suppressing the excessive release of inflammatory mediators, such as NO, IL‐1β, IL‐6, and TNF‐α (Chen et al. 2025). This mechanism may be associated with the inhibition of the overactivated Toll‐like receptor 4 (TLR4)/myeloid differentiation primary response 88 (Myd88)/NLR family pyrin domain containing 3 (NLRP3) signaling pathway (Chen et al. 2025). Therefore, CVP‐I and CVP‐II not only possess stable triple‐helix structures, but also demonstrate promising potential in terms of structural stability and anti‐inflammatory functions, making them suitable candidates for development as functional foods and gut immune modulators.

#### 3.5.4 XRD Analysis

**FIGURE 7:**
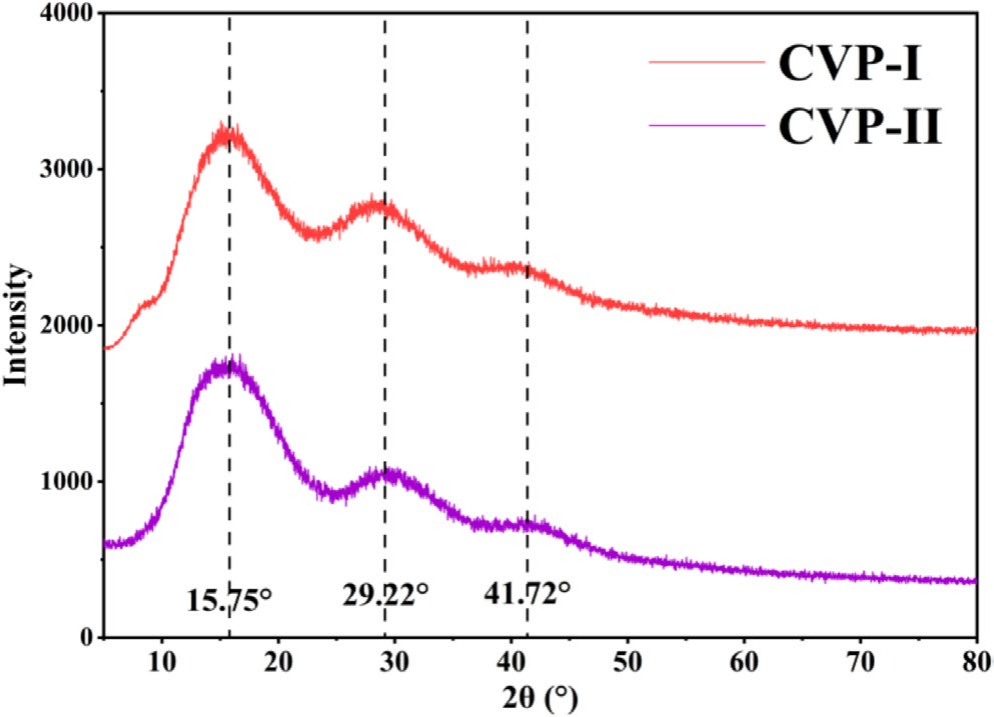
XRD spectra of CVP‐I and CVP‐II.

XRD is an important means for characterizing the crystalline properties of polysaccharides. As shown in Figure 7, CVP‐I and CVP‐II exhibit similar crystallization characteristics. Both polysaccharides show broad diffuse peaks near 2θ values of 15.75°, 29.22°, and 41.72°, indicating that their structures are primarily composed of amorphous, but also contain minor crystal regions (Fan et al. 2023). Notably, within the range of 50°–80°, the curves of CVP‐I and CVP‐II are relatively flat, further corroborating the amorphous structure of the polysaccharides (Liu et al. 2022). It can thus be inferred that both polysaccharides possess a structure consisting of the coexistence of crystal and amorphous phases, but with a low degree of crystallinity. Furthermore, numerous studies have reported that the physical properties of polysaccharides, such as flexibility, solubility, and expansibility, are directly determined by their crystalline structures (Jiang et al. 2025; Nuerxiati et al. 2024; Wang et al. 2019). The lower the crystallinity, the looser the arrangement of polysaccharide molecular chains, and the better the flexibility and solubility (Yu, Zhu, et al. 2024). Therefore, the low crystallinity of CVP‐I and CVP‐II contributes to their excellent flexibility, rapid solubility, and high expansibility, making them suitable as drug sustained‐release carriers to enhance drug stability and controlled release capabilities.

#### 3.5.5 Morphology Characteristics Analysis

**FIGURE 8:**
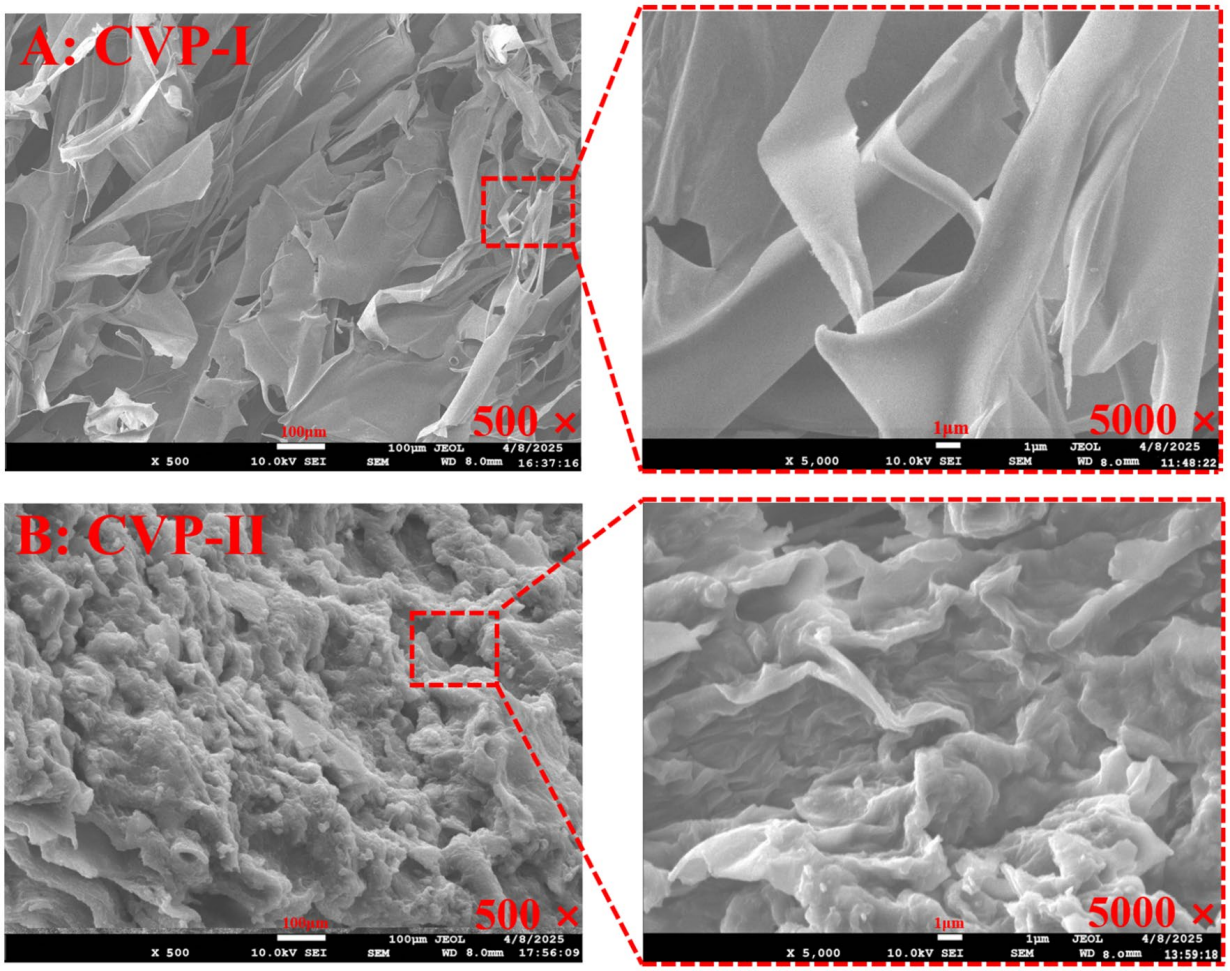
Surface morphology of CVP‐I (A) and CVP‐II (B) at 500× (scale bar: 100 μm) and 5000× (scale bar: 1 μm).

SEM can be used to observe the complex morphology and structural characteristics of polysaccharides. Figure 8 shows the SEM images of CVP‐I (Figure 8A) and CVP‐II (Figure 8B) at magnifications of 500 × and 5000 ×. The results demonstrate that the two polysaccharides exhibit significantly different micromorphological characteristics.

At low magnification (500 ×), CVP‐I presents a dense sheet‐like structure formed by the accumulation of irregular fibrous flakes, with distinct edges but displaying characteristics of tearing and fracture. Under high magnification (5000 ×), further observation reveals a relatively smooth surface without obvious pores, but with wavy and wrinkled undulations. This dense yet flexible morphology provides it with excellent hydration capacity and shear‐thinning properties, making it suitable for applications such as drug delivery and food thickening (Tian et al. 2024). In contrast, CVP‐II exhibits a typical spongy porous structure. At low magnification, numerous interconnected fibers and particles are visible, along with a large number of irregular pores, forming a three‐dimensional network structure. High‐magnification observation shows that the pore walls are thin, the surface is rough, and accompanied by irregular striated clustered protrusions, primarily aggregated of microfibers and particles. This loose and porous structure imparts rapid water absorption and swelling capacity, offering potential applications in fields such as tissue engineering scaffolds, wound dressings, and superabsorbent materials (Liu et al. 2022).

#### 3.5.6 Particle Size and Zeta Potential Analysis

**FIGURE 9:**
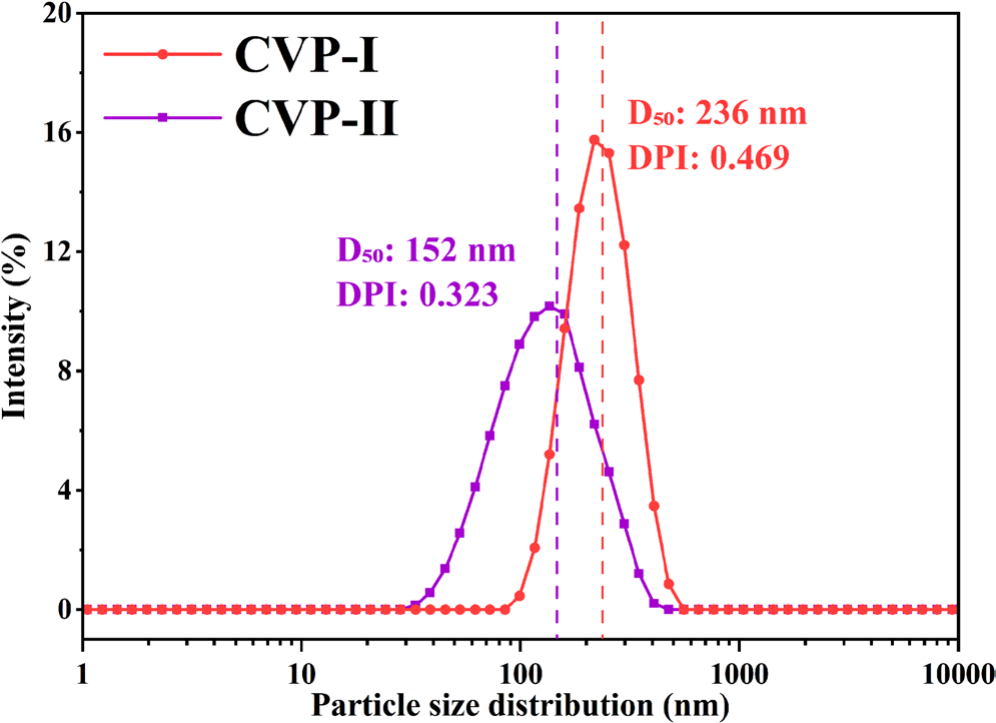
Particle size distribution curves of CVP‐I and CVP‐II.

Particle size and its distribution are important indicators for evaluating the uniformity and dispersibility of polysaccharides in aqueous solutions. As shown in Figure 9, the particle size distribution curves of CVP‐I and CVP‐II are relatively steep, indicating that both polysaccharides have a fairly uniform particle size distribution (Burken and Sommer 2024). Specifically, the particle size of CVP‐I is primarily distributed in the range of 99–476 nm, with an average particle size of 236 nm, while that of CVP‐II is concentrated in the range of 33–347 nm, with an average particle size of 152 nm. Notably, the polydispersity index (PDI) values of CVP‐I and CVP‐II are 0.469 and 0.323, respectively, both below 0.5, which suggests that both polysaccharides possess excellent dispersibility (Bu et al. 2024).

Zeta potential is commonly used to assess the stability of polysaccharides in aqueous environments. The higher the absolute value of the zeta potential, the stronger the electrostatic repulsion between polysaccharide particles, and the better the stability of the system (Griesser et al. 2017). The zeta potential of CVP‐II is −17.12 mV, which is significantly higher than that of the nearly neutral CVP‐I (0.03 mV) (*p* < 0.05), indicating superior stability of CVP‐II in solution. Furthermore, the stronger negative zeta potential may also contribute to enhancing the anti‐inflammatory activity of polysaccharides (Azari‐Anpara et al. 2023). The results demonstrate that both CVP‐I and CVP‐II exhibit good particle size uniformity and dispersibility, with CVP‐II showing better colloidal stability and potential anti‐inflammatory activity.

#### 3.5.7 Thermal Performance Analysis

**FIGURE 10:**
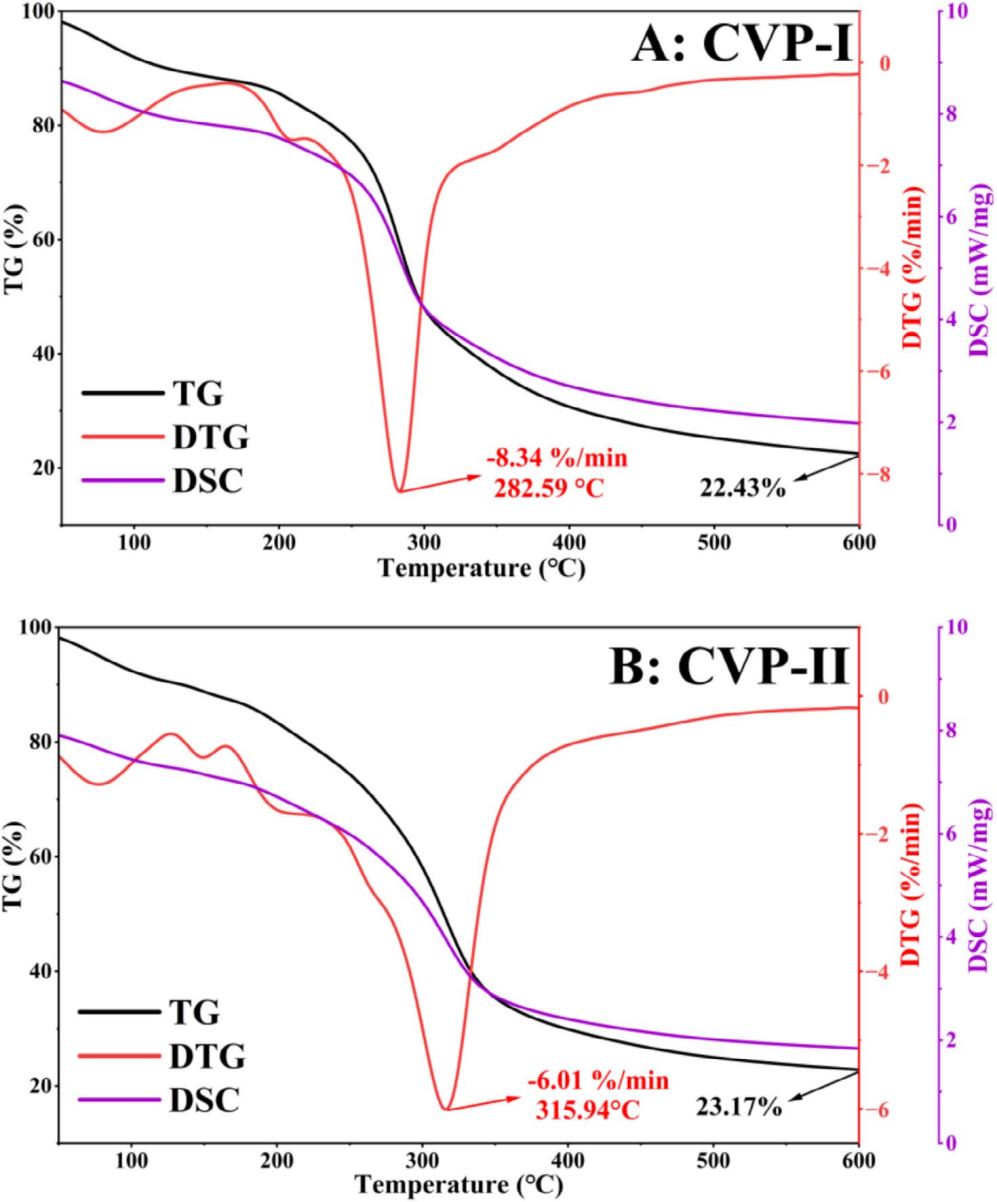
Thermal analysis curves (TG, DTG, and DSC) of CVP‐I (A) and CVP‐II (B).

Thermal stability analysis can quantitatively characterize the changes in polysaccharide mass over temperature and time during dehydration, decomposition, and oxidation processes. This study systematically investigated the thermal degradation characteristics of CVP‐I and CVP‐II using TG, DTG, and DSC (Figure 10). As shown in Figure 10A,B, the TG curves of CVP‐I and CVP‐II exhibited similar shapes, both showing three distinct weight‐loss stages in the temperature range of 50°C–600°C. The first stage (50°C–158°C) primarily resulted from the removal of adsorbed water and crystalline water (Liu et al. 2022). The second stage (191°C–436°C) was mainly due to depolymerization and degradation of the polysaccharides, and the mass loss rate at this stage exceeded 50% of the initial weight (Li et al. 2022). The third stage (461°C–600°C) showed a relatively slow degradation rate and eventually stabilizes (Nuerxiati et al. 2024). Notably, the residual mass fraction of CVP‐II (23.17%) was higher than that of CVP‐I (22.43%).

The DTG curves revealed that the maximum decomposition rate of CVP‐II (−6.01%/min) was lower than that of CVP‐I (−8.34%/min), and its maximum decomposition temperature (315.94°C) was significantly higher than that of CVP‐I (282.59°C) by 33.35°C. DSC test results indicated that both polysaccharides displayed characteristic peaks of glass transition in the 84°C–137°C range, demonstrating the motion of their amorphous polysaccharide molecular chains, which is consistent with the XRD analysis (Yan et al. 2021). Additionally, within the range of 192°C–328°C, we observed exothermic peaks in the curve associated with the chemical decomposition of polysaccharides (Abuduwaili et al. 2022).

The comprehensive analysis of TG, DTG, and DSC curves demonstrates that both polysaccharides exhibit excellent thermal stability below 280°C, with CVP‐II displaying superior resistance to thermal degradation. This property gives it broad application potential in high‐temperature sterilization of food, sustained‐release pharmaceutical formulations, heat‐resistant biomaterials, and high‐temperature processing of cosmetics (Cui et al. 2025).

#### 3.5.8 Methylation Analysis

Methylation analysis provided critical information for elucidating the glycosidic linkage patterns of CVP‐I and CVP‐II (Table 5). Comparison with the CCRC PMAA reference database revealed that both polysaccharides contain five monosaccharide residues: Fuc, Rha, Ara, Man, and Glc and are linked through 10 different glycosidic bonds.

**TABLE 5:** Methylation analysis results of CVP‐I and CVP‐II.

Sugar residues		Methylated sugars	Retention time (min)	Linkage patterns	Mass fragments (m/z)	Molar ratio %
CVP‐I	Fuc	2‐Me_1_‐Fuc	39.8	→3,4)‐Fuc*p*‐(1→	131, 88, 101, 118, 160, 175	5.45
		2,3‐Me_2_‐Fuc	31.2	→4)‐Fuc*p*‐(1→	118, 131, 89, 102, 162, 175	32.57
		2,3,4‐Me_3_‐Fuc	24.5	T‐Fuc*p*	72, 89, 101, 115, 118, 131, 161	4.49
	Rha	2‐Me_1_‐Rha	46.5	→2,4)‐Rha*p*‐(1→	131, 88, 101, 130, 190	20.27
		3,4‐Me_2_‐Rha	25.8	T‐Rha*p*	72, 89, 101, 115, 118, 131	5.32
	Ara	2,4,5‐Me_3_‐Ara	24.2	T‐Ara*f*	59, 71, 87, 102, 118, 129, 161	18.11
	Man	2,3,4,6‐Me_4_‐Man	29.5	Man*p*‐(1→	102, 118, 129, 145, 161, 205	3.17
		2,4‐Me_2_‐Man	44.5	→3,6)‐Man*p*‐(1→	118, 129, 189, 233	1.58
	Glc	2,3,6‐Me_3_‐Glc	37.5	→4)‐Glc*p*‐(1→	118, 129, 143, 162, 173, 233	6.85
		2,3,4,6‐Me_4_‐Glc	28.8	Glc*p*‐(1→	102, 118, 129, 145, 161, 205	2.19
CVP‐II	Fuc	2‐Me_1_‐Fuc	39.8	→3,4)‐Fuc*p*‐(1→	131, 88, 101, 118, 160, 175	4.34
		2,3‐Me_2_‐Fuc	31.2	→4)‐Fuc*p*‐(1→	118, 131, 89, 102, 162, 175	3.72
		2,3,4‐Me_3_‐Fuc	24.5	T‐Fuc*p*	72, 89, 101, 115, 118, 131, 161	11.15
	Rha	2‐Me_1_‐Rha	46.5	→2,4)‐Rha*p*‐(1→	131, 88, 101, 130, 190	29.41
		3,4‐Me_2_‐Rha	25.8	T‐Rha*p*	72, 89, 101, 115, 118, 131	4.19
	Ara	2,4,5‐Me_3_‐Ara	24.2	T‐Ara*f*	59, 71, 87, 102, 118, 129, 161	15.83
	Man	2,3,4,6‐Me_4_‐Man	29.5	Man*p*‐(1→	102, 118, 129, 145, 161, 205	3.64
		2,4‐Me_2_‐Man	44.5	→3,6)‐Man*p*‐(1→	118, 129, 189, 233	6.22
	Glc	2,3,6‐Me_3_‐Glc	37.5	→4)‐Glc*p*‐(1→	118, 129, 143, 162, 173, 233	19.12
		2,3,4,6‐Me_4_‐Glc	28.8	Glc*p*‐(1→	102, 118, 129, 145, 161, 205	2.38

In CVP‐I, Fuc residues were the most abundant, including →3,4)‐Fuc*p*‐(1 → (5.45%), →4)‐Fuc*p*‐(1 → (32.57%), and T‐Fuc*p* (4.49%). These were followed by Rha residues, comprising →2,4)‐Rha*p*‐(1 → (20.27%) and T‐Rha*p* (5.32%). Ara residues were primarily present as T‐Ara*f* (18.11%). Glc residues included →4)‐Glc*p*‐(1 → (6.85%) and Glc*p*‐(1 → (2.19%), while Man residues consisted of Man*p*‐(1 → (3.17%) and →3,6)‐Man*p*‐(1 → (1.58%). These results indicate that the backbone of CVP‐I is mainly composed of →4)‐Fuc*p*‐(1 → and →2,4)‐Rha*p*‐(1→, with T‐Ara*f* as the predominant terminal residue, while the remaining residues are likely distributed in the side chains.

In contrast, CVP‐II is primarily composed of Rha residues (→2,4)‐Rha*p*‐(1 → (29.41%) and T‐Rha*p* (4.19%). These are followed by Glc residues (→4)‐Glc*p*‐(1 → (19.12%) and Glc*p*‐(1 → (2.38%), Fuc residues (→3,4)‐Fuc*p*‐(1 → (4.34%), →4)‐Fuc*p*‐(1 → (3.72%), and T‐Fuc*p* (11.15%), Ara residues (T‐Ara*f* (15.83%), and Man residues (Man*p*‐(1 → (3.64%) and →3,6)‐Man*p*‐(1 → (6.22%). This suggests that the backbone of CVP‐II is mainly constituted by →2,4)‐Rha*p*‐(1 → and →4)‐Glc*p*‐(1→, with T‐Fuc*p* and T‐Ara*f* as the major terminal residues, while the other linkages are likely located in the side chains.

Furthermore, the ten structural units in CVP‐I, namely →3,4)‐Fuc*p*‐(1→, →4)‐Fuc*p*‐(1→, T‐Fuc*p*, →2,4)‐Rha*p*‐(1→, T‐Rha*p*, T‐Ara*f*, Man*p*‐(1→, →3,6)‐Man*p*‐(1→, →4)‐Glc*p*‐(1→, and Glc*p*‐(1→, have a molar ratio of 3.45:20.61:2.84:12.83:3.37:11.46:2.01:1:4.34:1.39, while the molar ratio of the corresponding structural units in CVP‐II is 1.82:1.56:4.68:12.36:1.76:6.65:1.53:2.61:8.03:1. These molar ratios are consistent with the monosaccharide composition results of the two polysaccharides. Notably, both CVP‐I and CVP‐II exhibit a high proportion of terminal residues (27.92% and 31.17%, respectively), indicating that both polysaccharides possess long, straight‐chain structures with low branching (Ji et al. 2018). In conclusion, CVP‐I and CVP‐II exhibit significantly distinct glycosidic linkage patterns, reflecting their unique structural architectures.

#### 3.5.9 ^1^H NMR and ^13^C NMR Spectra Analysis

**FIGURE 11:**
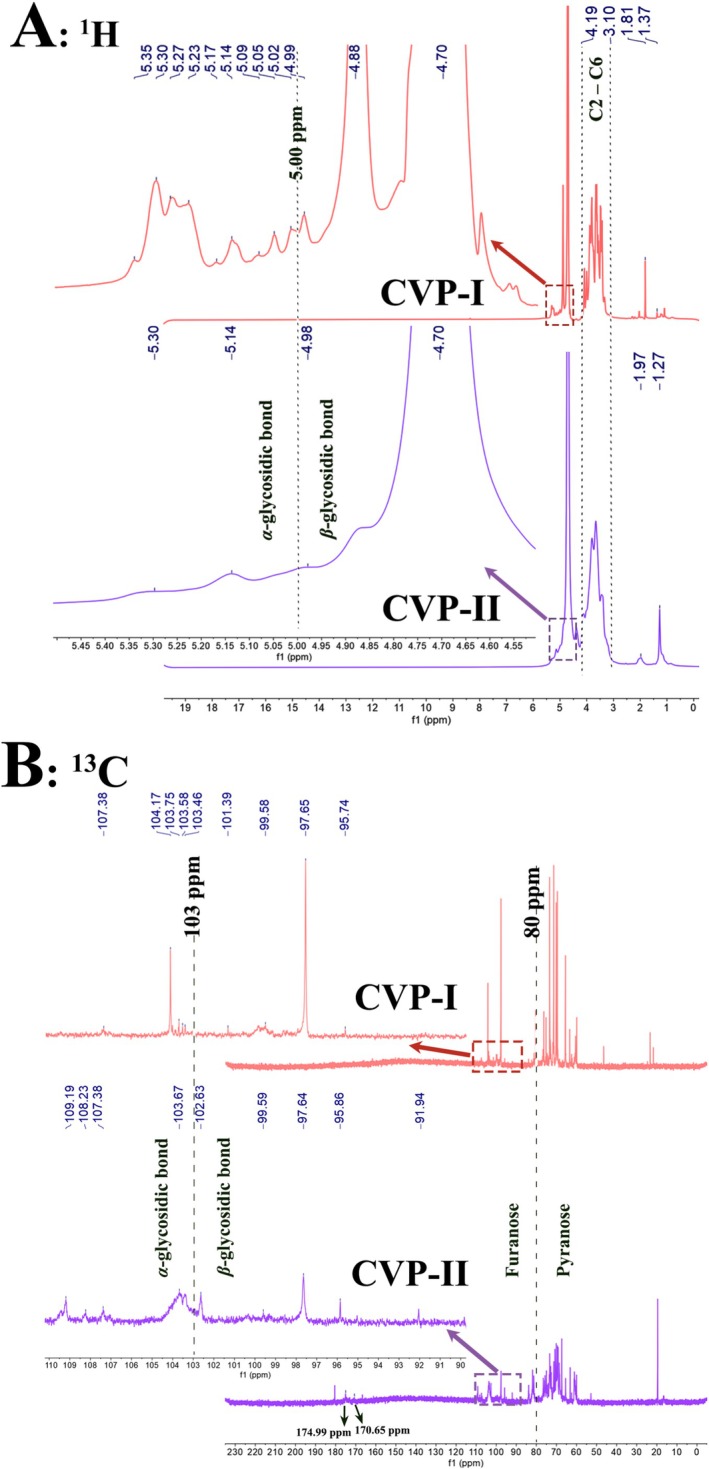
NMR spectra of CVP‐I and CVP‐II. (A) ^1^H NMR spectra, (B) ^13^C NMR spectra.

NMR is a powerful tool for elucidating the structure of polysaccharides, enabling accurate identification of their isomeric configurations (α‐ or β‐type), glycosidic linkage patterns, and sugar residue sequences. As shown in Figure 11A, in the ^1^H NMR spectra of CVP‐I and CVP‐II, the δ 4.70 ppm signal corresponds to the D_2_O solvent peak (Chen et al. 2023). In the anomeric proton region (δ 4.8–5.5 ppm), CVP‐I exhibits 11 distinct anomeric proton signals within the range of δ 4.88–5.35 ppm, indicating that it contains at least 11 different types of sugar residues (Al‐Wraikat et al. 2022). In contrast, CVP‐II displays only three anomeric proton signals at δ 4.98, 5.14, and 5.30 ppm, corresponding to three distinct sugar residues. Typically, the chemical shift of anomeric proton in α‐type sugar residues is greater than δ 5 ppm, while that of β‐type is less than δ 5 ppm (Fan et al. 2023; Sun et al. 2024). The anomeric proton signals of both CVP‐I and CVP‐II are distributed on both sides of δ 5.0 ppm, confirming the coexistence of α‐ and β‐configurations in both polysaccharides, which is consistent with the FT‐IR spectra analysis. Additionally, signals in the range of δ 3.10–4.19 ppm can be attributed to the C2‐C6 protons on the polysaccharide rings (Liu et al. 2024). Notably, the characteristic peaks at δ 1.37 ppm for CVP‐I and δ 1.27 ppm for CVP‐II both correspond to the methyl protons of Rha, while the signals appearing at δ 1.81 ppm for CVP‐I and δ 1.97 ppm for CVP‐II may originate from the methyl protons of the O‐acetyl groups substituted on the sugar residues (Wang, Zeng, et al. 2023).

The ^13^C NMR spectra (Figure 11B) of CVP‐I and CVP‐II, measured at the same temperature, show 9 signals detected within the typical anomeric carbon region (δ 90–110 ppm) for both (Liu et al. 2021). Among these, the anomeric carbon signals of CVP‐I are distributed between δ 95.74–107.38 ppm, while the signals of CVP‐II are located between δ 91.94–109.19 ppm. The chemical shifts of both polysaccharides are distributed on either side of δ 103 ppm, further confirming the coexistence of α‐ and β‐configurations (Lin et al. 2016). This result aligns with the FT‐IR spectra analysis and ^1^H NMR spectra analysis. It should be noted that the significant discrepancy between the number of anomeric proton signals (*n* = 11) in the ^1^H NMR spectrum and the number of anomeric carbon signals (*n* = 9) in the ^13^C NMR spectrum of CVP‐I may be attributed to signal overlap, low abundance of certain residues, or insufficient relaxation times for quantitative acquisition in the ^13^C NMR analysis. Typically, the C3 and C5 resonance signals of furanose appear above δ 80 ppm, while those of pyranose are below this value (Xiong et al. 2023). The C2‐C6 signals of both CVP‐I and CVP‐II are distributed around δ 80 ppm, indicating that both contain both furanose and pyranose structures, with pyranose predominating. Notably, the signals in the range of δ 170–180 ppm are characteristic peaks of uronic acid (Zhou et al. 2025). No such signals were detected in CVP‐I, whereas CVP‐II exhibited two distinct peaks at δ 170.65 and 174.99 ppm, suggesting the presence of trace amounts of uronic acid components. This finding is consistent with the uronic acid content in Table 4 and the UV spectra analysis. Additionally, the strong signal at δ 103.67 ppm in CVP‐II corresponds to C‐1 of the (1 → 4)‐linked β‐D‐glucopyranose units, while the low‐field‐shifted signal at δ 101.39 ppm in CVP‐I may arise from the C‐1 of another structural unit (Chen et al. 2021; Lin et al. 2023). This result corroborates the findings from the methylation analysis.

In addition, the structural characteristics of CVP‐I and CVP‐II importantly influence their functional properties and anti‐inflammatory activity. Among these, the CVP‐I structure is complex, comprising 11 distinct sugar residues, diverse glycosidic bond linkages, and O‐acetyl groups attached to the sugar residues. These structural features may enhance binding affinity with macrophage membrane receptors by modulating the intestinal immune microenvironment to form multiple receptor‐binding sites, thereby suppressing pro‐inflammatory cytokine release and promoting tissue repair (Chen et al. 2023; Yuan et al. 2022). In contrast, CVP‐II contains uronic acid components and exhibits fewer anomeric proton signals. It may exert anti‐inflammatory effects by enhancing macrophage function through chelation of metal ions, regulation of complement activation, or direct binding to immune cell receptors (Kang et al. 2023). Similarly, the presence of (1 → 4)‐linked β‐D‐glucopyranose units in the CVP‐II structure, which are speculated to potentially participate in inhibiting NF‐κB and MAPK signaling pathways, thereby downregulating overexpressed proinflammatory mediators (Jin et al. 2021; Qiu et al. 2025; Uhliariková et al. 2020). Notably, both polysaccharides primarily exist in the form of pyranose rings and exhibit the characteristic coexistence of α‐ and β‐configurations. This structure might help maintain conformational stability and good solubility under physiological conditions, thereby facilitating recognition by pattern recognition receptors on the surface of immune cells, such as macrophages and dendritic cells, subsequently promoting effective activation of the immune response and regulating the release of inflammatory mediators (Zhou et al. 2020).

### 3.6 Anti‐Inflammatory Activity Analysis

#### 3.6.1 Impact of CVP‐I and CVP‐II on the Viability of RAW264.7 Cells

**FIGURE 12:**
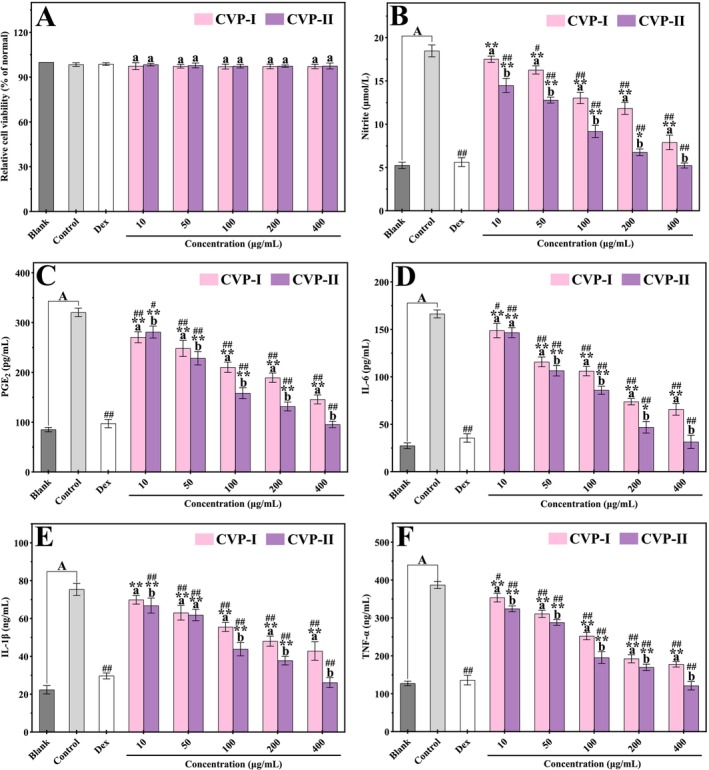
CVP‐I and CVP‐II cytotoxic effects on RAW264.7 macrophages (A). Inhibitory effects of CVP‐I and CVP‐II on the production of NO (B), PGE_2_ (C), IL‐6 (D), IL‐1β (E), and TNF‐α (F) in LPS‐induced RAW264.7 macrophages. (A) *p* < 0.05; different letters (a, b) indicate significant differences between samples at the same concentration (*p* < 0.05); *p < 0.05, **p < 0.01, compared to blank group; #*p* < 0.05, ##*p* < 0.01, compared to control group.

The study investigated the effects of different concentrations (10, 50, 100, 200, and 400 μg/mL) of CVP‐I and CVP‐II on the viability of LPS‐induced RAW264.7 macrophages. As shown in Figure 12A, within the tested concentration range, neither CVP‐I nor CVP‐II caused significant changes in cell viability compared to the blank group (*p* > 0.05). Furthermore, no statistically significant differences were observed between the two polysaccharides at identical concentrations (*p* > 0.05). Similarly, both the control and positive control groups exhibited no significant alterations in cell viability compared to the blank group (*p* > 0.05). These results indicate that neither CVP‐I, CVP‐II, LPS, nor dexamethasone exhibited cytotoxic effects on RAW264.7 macrophages.

#### 3.6.2 Impact of CVP‐I and CVP‐II on LPS‐Induced NO and PGE_2_ Production

LPS‐induced NO and PGE_2_ are key inflammatory mediators in immune responses. NO is synthesized from L‐arginine under catalysis by nitric oxide synthase (NOS) and plays a significant role in inflammatory signaling (Zhou et al. 2020). Excessive NO production is associated with the pathogenesis of various inflammatory diseases, making it an important marker for assessing cellular inflammatory status and the efficacy of anti‐inflammatory drugs (Sun et al. 2020). Similarly, PGE_2_ is produced through the metabolism of arachidonic acid mediated by cyclooxygenase (COX) and participates in cellular signaling processes such as inflammatory responses and tumor metastasis (Xiong et al. 2017).

As shown in Figure 12B,C, compared with the blank and positive control groups, LPS stimulation significantly increased the levels of NO and PGE_2_ in RAW264.7 macrophages (*p* < 0.01), indicating the successful establishment of an inflammatory cell model (Wei et al. 2021). After treatment with CVP‐I and CVP‐II, the levels of both NO and PGE_2_ in cells decreased in a concentration‐dependent manner. At the same concentrations, the inhibitory effects of the two polysaccharides showed significant differences (*p* < 0.05), with CVP‐II exhibiting a stronger inhibitory effect (*p* < 0.05). Specifically, within the concentration range of 10–400 μg/mL, the inhibition rates of CVP‐II on NO (71.68% ± 0.96%) and PGE_2_ (70.22% ± 1.27%) were higher than those of CVP‐I (57.23% ± 1.37% and 54.54% ± 1.68%, respectively). Notably, at a concentration of 400 μg/mL, CVP‐II reduced NO and PGE_2_ levels to 5.23 ± 0.16 μmol/L and 95.42 ± 1.53 pg/mL, respectively, both below those of the positive control group (5.62 ± 0.31 μmol/L and 97.29 ± 2.83 pg/mL, respectively), and showed no significant difference compared to the blank group (*p* > 0.05). These results indicate that although both polysaccharides possess anti‐inflammatory activity, CVP‐II demonstrates superior efficacy, which may be related to its negatively charged groups and unique molecular conformation (Chen et al. 2023).

#### 3.6.3 Impact of CVP‐I and CVP‐II on LPS‐Induced Pro‐Inflammatory Cytokine Production

The excessive production of pro‐inflammatory cytokines (IL‐6, IL‐1β, and TNF‐α) by immune cells in activated macrophages can trigger severe inflammation‐related diseases. These cytokines exacerbate immune responses by activating multiple inflammatory pathways (Azab et al. 2016). Among these, IL‐6 acts as an endogenous pyrogen and regulates the production of acute‐phase proteins (Xiong et al. 2017). IL‐1β plays a pivotal role in the early stages of inflammation (Oliveira et al. 2017). TNF‐α exacerbates the inflammatory response by enhancing neutrophil and lymphocyte activity and promoting the release of other inflammatory mediators (Mehanna et al. 2021).

As shown in Figure 12D–F, compared with the blank group, LPS stimulation significantly upregulated the secretion of IL‐6, IL‐1β, and TNF‐α (by 5.08‐fold, 2.37‐fold, and 2.04‐fold, respectively) (*p* < 0.05), indicating that LPS induces a severe inflammatory response. This response may be associated with an imbalance in cytokine levels and may involve the regulation of the NF‐κB/MAPK signaling pathway (Li et al. 2021). Notably, both CVP‐I and CVP‐II suppressed cytokine release in a dose‐dependent manner, with significant differences in their inhibitory effects observed at the same concentrations (*p* < 0.05). Within the concentration range of 10–400 μg/mL, the inhibition rates of CVP‐II on IL‐6 (Figure 12D), IL‐1β (Figure 12E), and TNF‐α (Figure 12F) were 81.09% ± 2.06%, 64.45% ± 1.77%, and 68.73% ± 1.63%, respectively, all higher than those of CVP‐I (60.43% ± 1.83%, 41.66% ± 2.64%, and 54.09% ± 1.08%, respectively). Furthermore, at a concentration of 400 μg/mL, CVP‐II restored the levels of the three pro‐inflammatory cytokines (31.45 ± 0.32 pg/mL, 26.09 ± 0.21 ng/mL, and 121.04 ± 0.55 ng/mL, respectively) to levels comparable to those of the blank group (27.33 ± 0.11 pg/mL, 22.35 ± 0.14 ng/mL, and 127.13 ± 0.28 ng/mL, respectively), and all were lower than those in the positive control group (35.56 ± 0.19 pg/mL, 29.67 ± 0.12 ng/mL, and 135.88 ± 0.64 ng/mL, respectively).

Overall, CVP‐II exhibited the strongest inhibitory effect on all three pro‐inflammatory cytokines. Based on this, it is speculated that this may stem from the negatively charged groups present in CVP‐II, such as hydroxyl and sulfate groups, which facilitate its interaction with inflammatory signaling molecules like NF‐κB and cyclooxygenase‐2 (COX‐2), thereby more effectively suppressing the release of pro‐inflammatory cytokines (Ma et al. 2020). To validate this conclusion, our subsequent research will integrate NF‐κB/MAPK pathway analysis, TLR4 antibody blockade experiments, and animal inflammation models to further explore its structure–activity relationship and molecular mechanisms.

## 4 Conclusion

In summary, this study successfully extracted polysaccharides from *C. vaginatum* using the UAE method, and optimized the UAE process parameters through single‐factor experiments and RSM. Under optimal conditions, the polysaccharide yield from UAE is significantly higher than that from HWE. Following separation and purification, two homogeneous polysaccharides, CVP‐I and CVP‐II, were obtained. Based on comprehensive chemical composition analysis, methylation analysis, and NMR spectra analysis, the backbone of CVP‐I is primarily composed of Fuc and Rha residues, while the side chains and terminal ends consist of Ara, Man, and Glc residues. In contrast, the backbone of CVP‐II mainly consists of Rha and Glc residues, with side chains and terminal residues including Fuc, Ara, and Man. Through multiple characterization methods such as UV, FT‐IR, Congo red assay, XRD, SEM, particle size and zeta potential analysis, TG, DTG, and DSC, it has been systematically revealed that the two polysaccharides possess distinct physicochemical properties and structural characteristics. Anti‐inflammatory activity assays demonstrated that CVP‐I and CVP‐II significantly inhibited the secretion of NO, PGE_2_, and pro‐inflammatory cytokines (IL‐6, IL‐1β, and TNF‐α) in LPS‐induced RAW264.7 macrophages, exhibiting excellent anti‐inflammatory activity. Among them, CVP‐II displayed the most pronounced anti‐inflammatory effect. These findings indicate that CVP components may serve as potential candidates for developing novel immunomodulatory functional foods. Future research will focus on CVP‐II, systematically analyzing its structural characteristics to explore the relationship between its structure or composition and anti‐inflammatory activity, aiming to provide a theoretical basis for its development and application in fields such as anti‐inflammatory functional foods and natural nutritional supplements.

## Author Contributions

**Conghui Ren:** methodology, visualization, conceptualization. **Yuhao Cui:** writing – original draft, validation, software. **Junlong Wang:** writing‐review and editing, formal analysis, funding acquisition. **Qingyou Fu:** methodology, conceptualization. **Shenghui Chen:** software, visualization. **Jie Li:** data curation, investigation. **Yonggang Lin:** resources, supervision, project administration.

## Funding

This work was supported by the Xinjiang Talent Development Fund Scientific Research Innovation Platform Talent Team Support Program Project, China (Project No. XJRCSWZTD2025‐11).

## Conflicts of Interest

The authors declare no conflicts of interest.

## Supporting information


**Figure S1:** Contour plots of factor interactions.
**Figure S2:** Mw curves of CVP‐I and CVP‐II.

## Data Availability

The data that support the findings of this study are available on request from the corresponding author. The data are not publicly available due to privacy or ethical restrictions.
