# Supplementary material for: Extraction, Structural Characterization and Anti‐Inflammatory Activity of Polysaccharides From Conioselinum vaginatum
Source: Food Sci Nutr. 2026 Apr 26;14(5):e71827. doi: 10.1002/fsn3.71827 (PMC13111767; doi:10.1002/fsn3.71827)
Supplement: Supplementary file 1 — Figure S1: Contour plots of factor interactions. Figure S2: Mw curves of CVP‐I and CVP‐II. [file FSN3-14-e71827-s001.docx]

**Support Information**

| 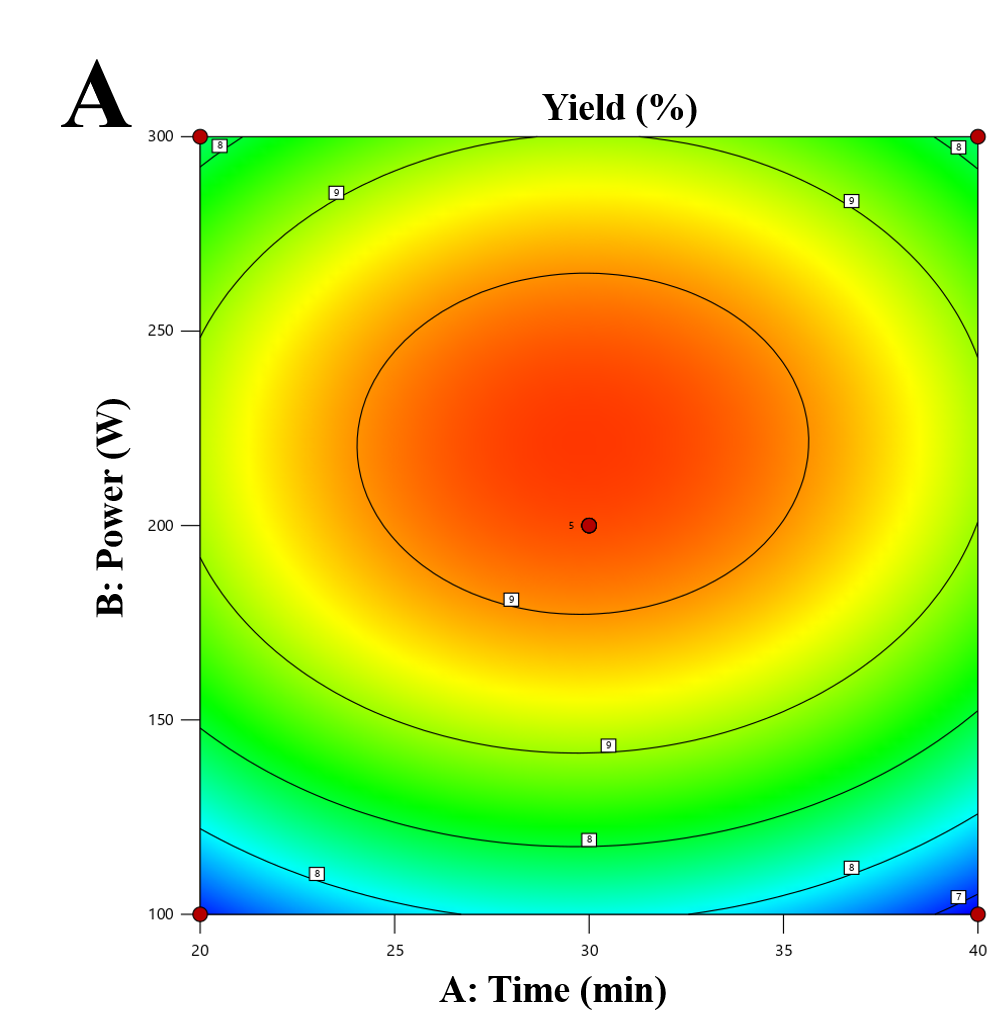 | 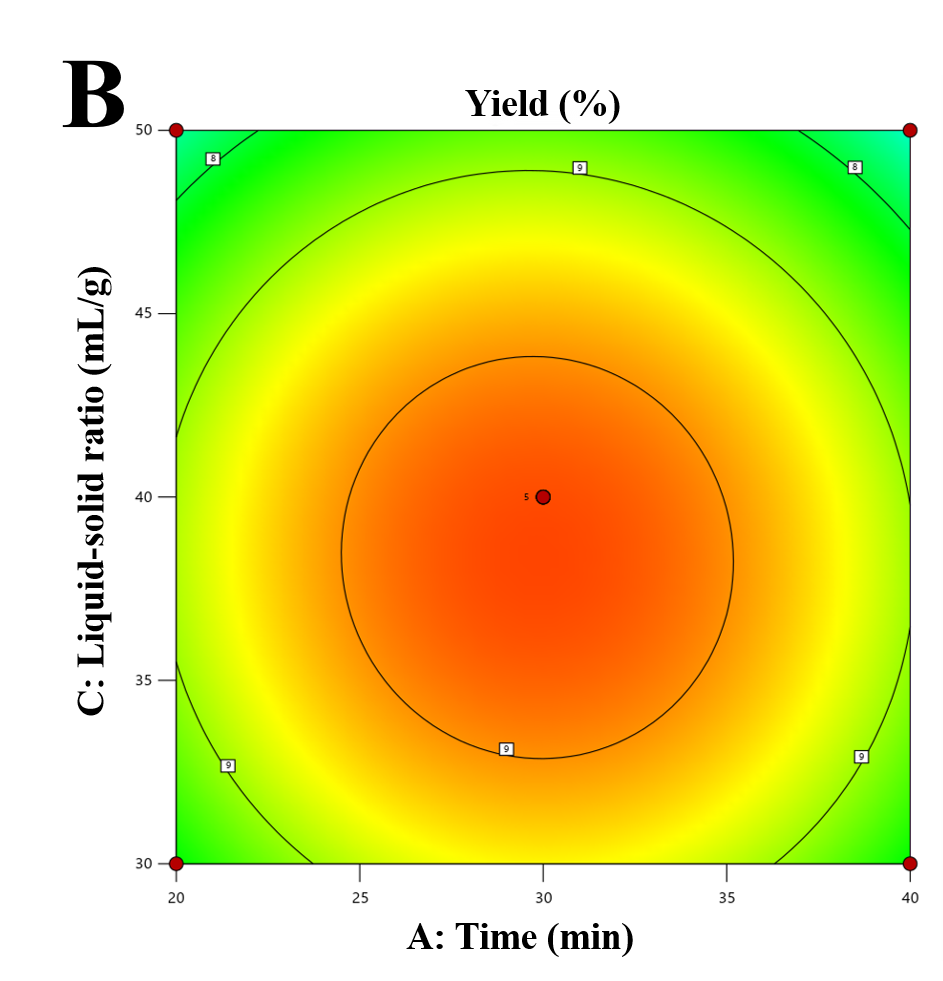 |
| --- | --- |
| 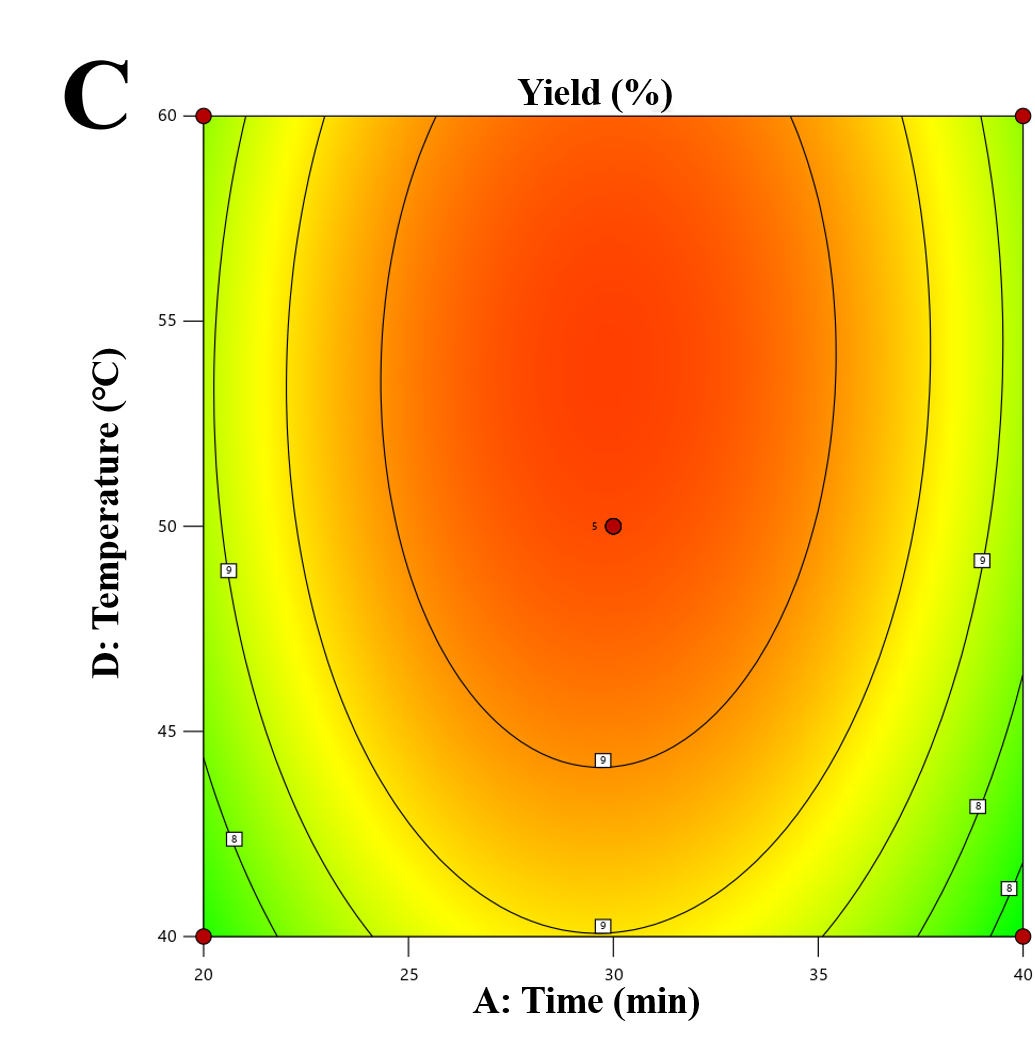 | 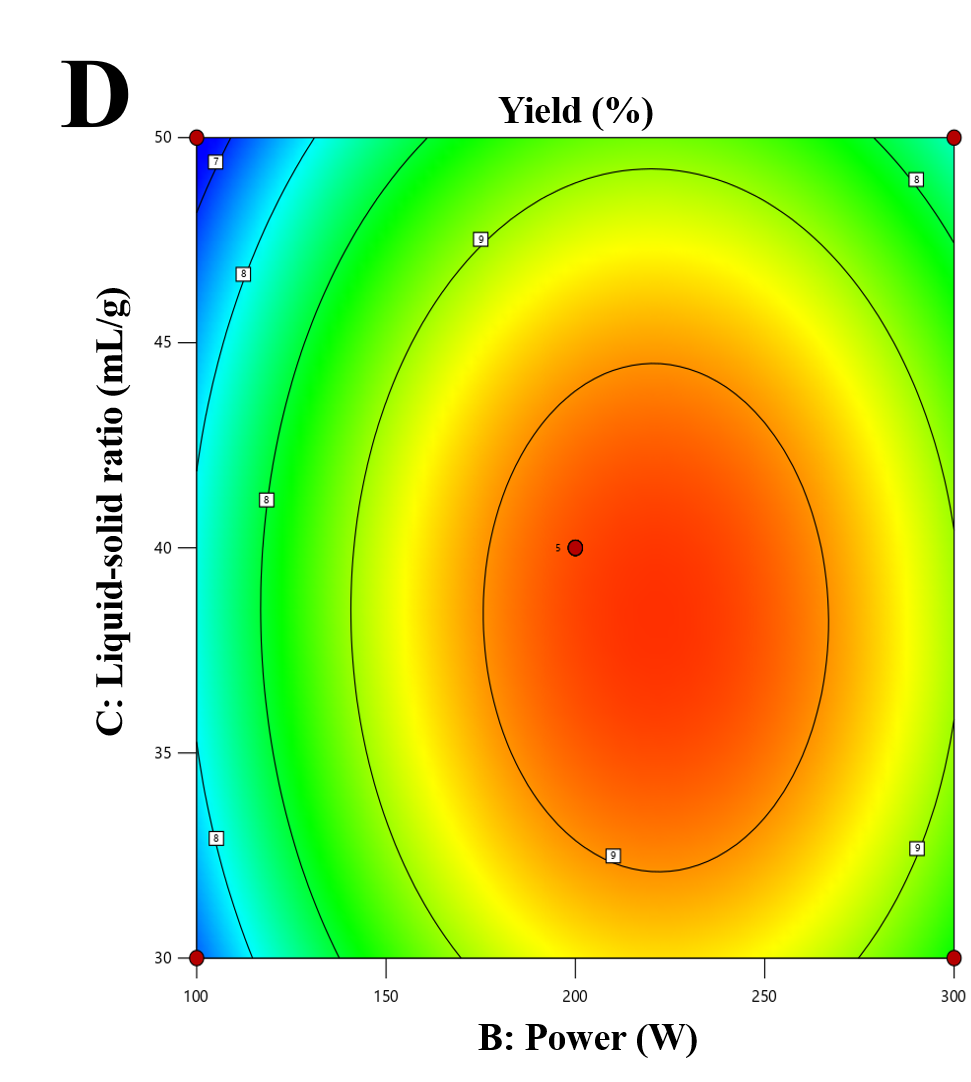 |
| 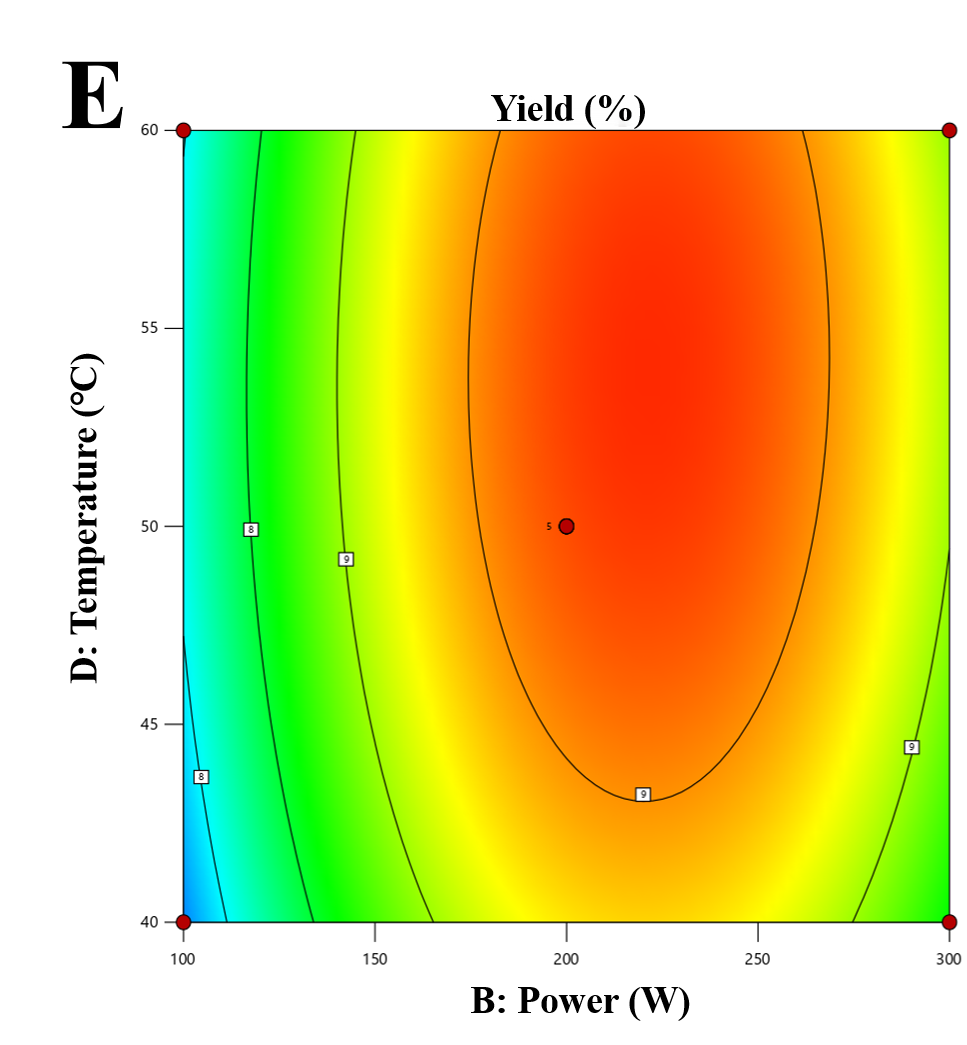 | 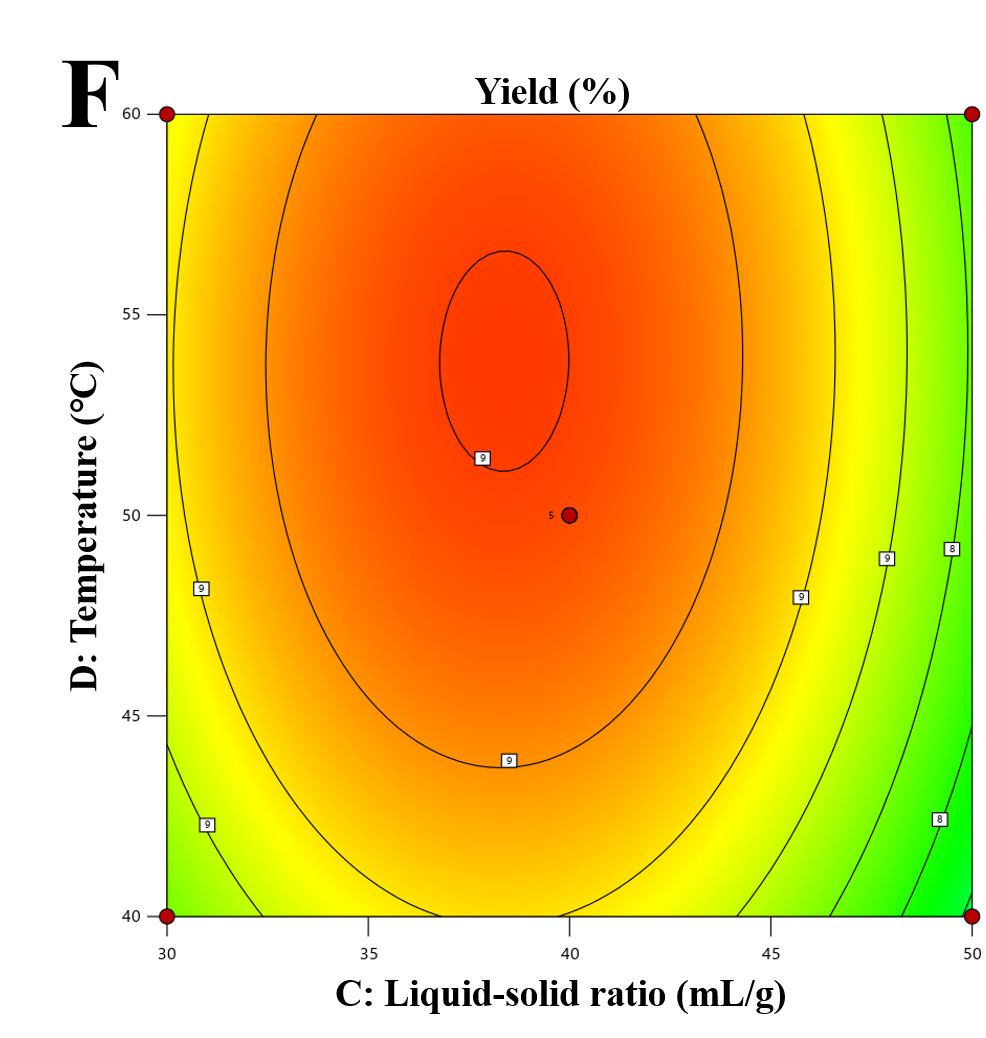 |

**Figure S1.** Contour plots of factor interactions.

**
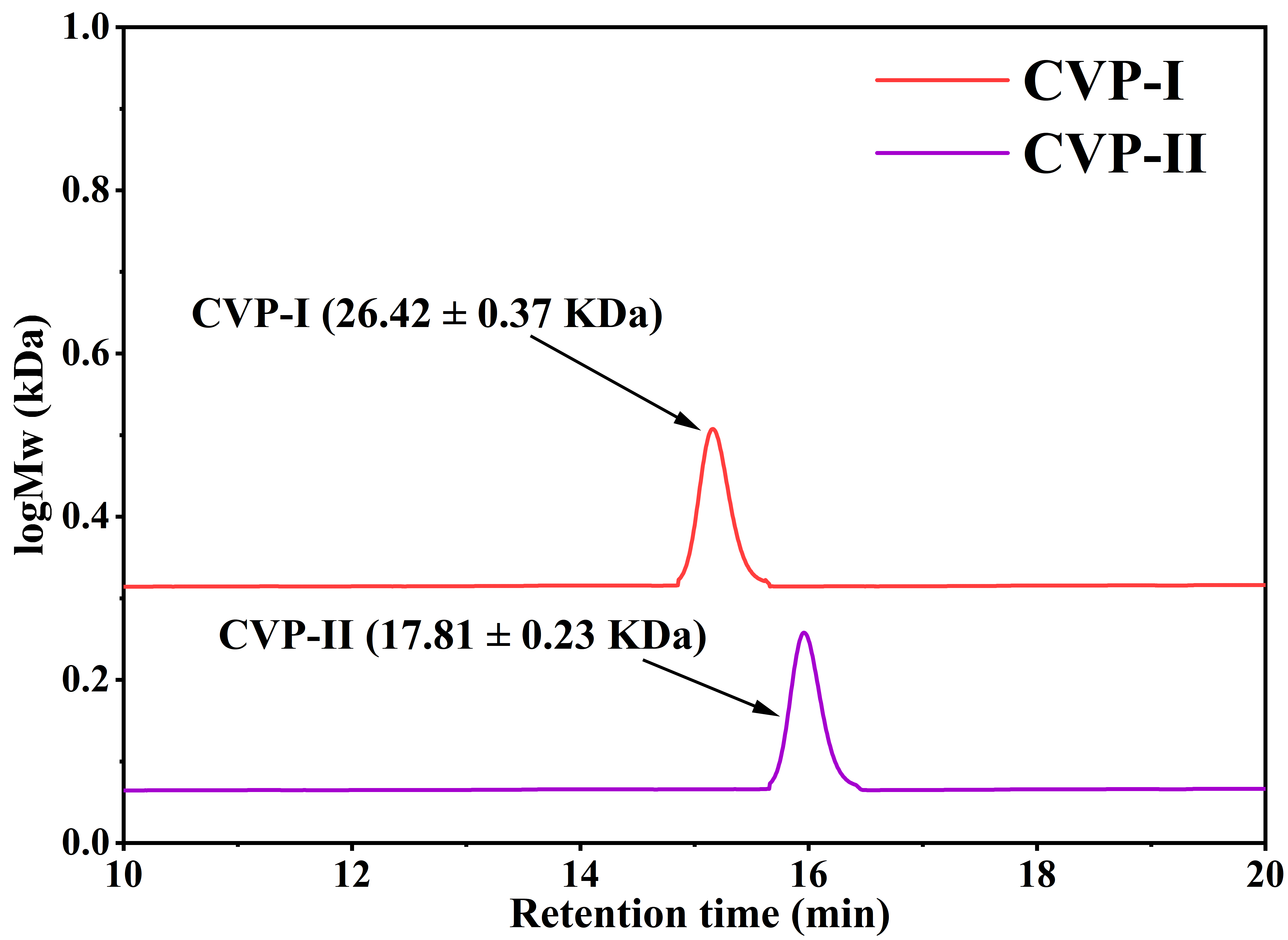
**

**Figure S2.** Mw curves of CVP-I and CVP-II.

**Glucose standard curve:**

**
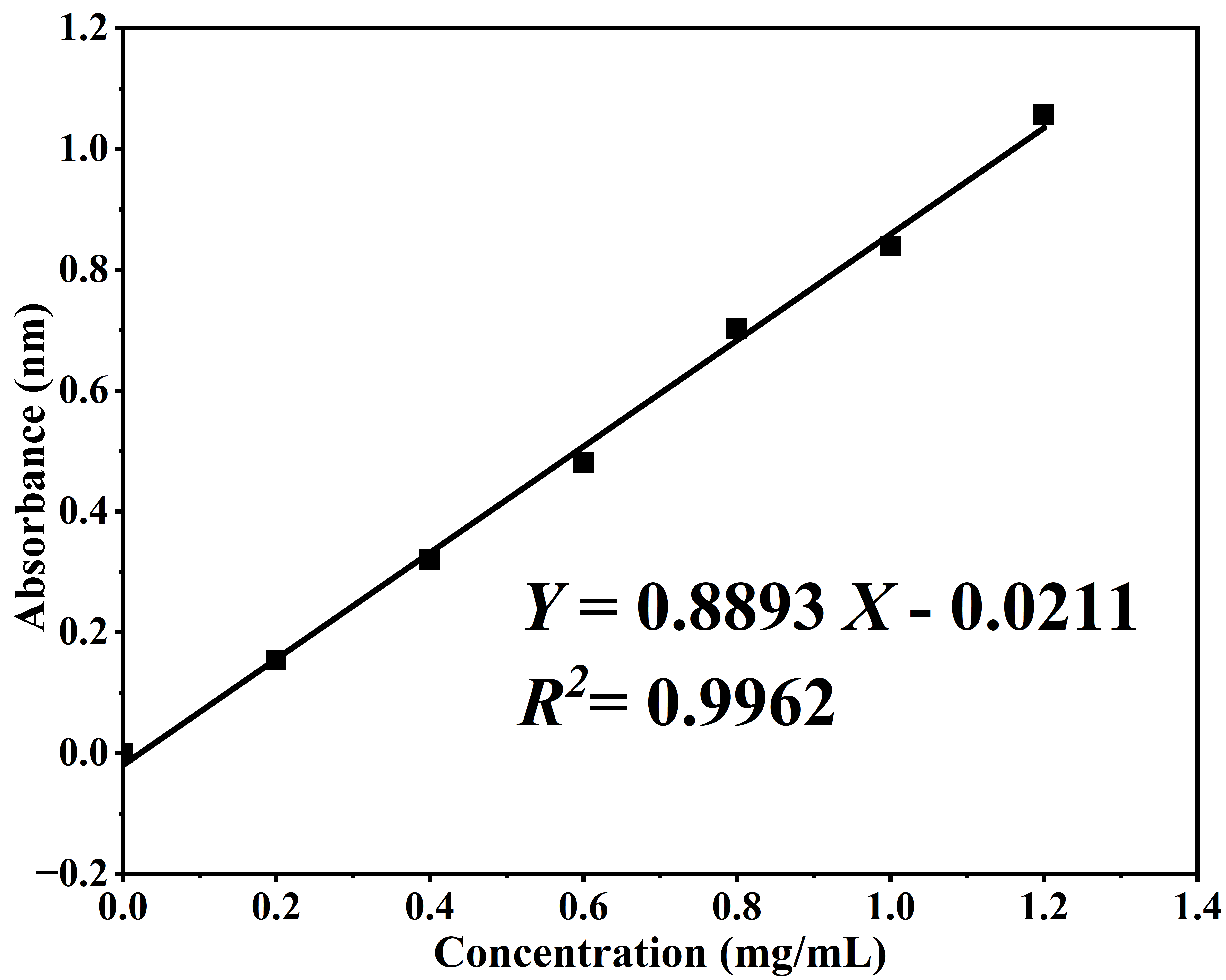
**

**Bovine serum albumin standard curve:**

**
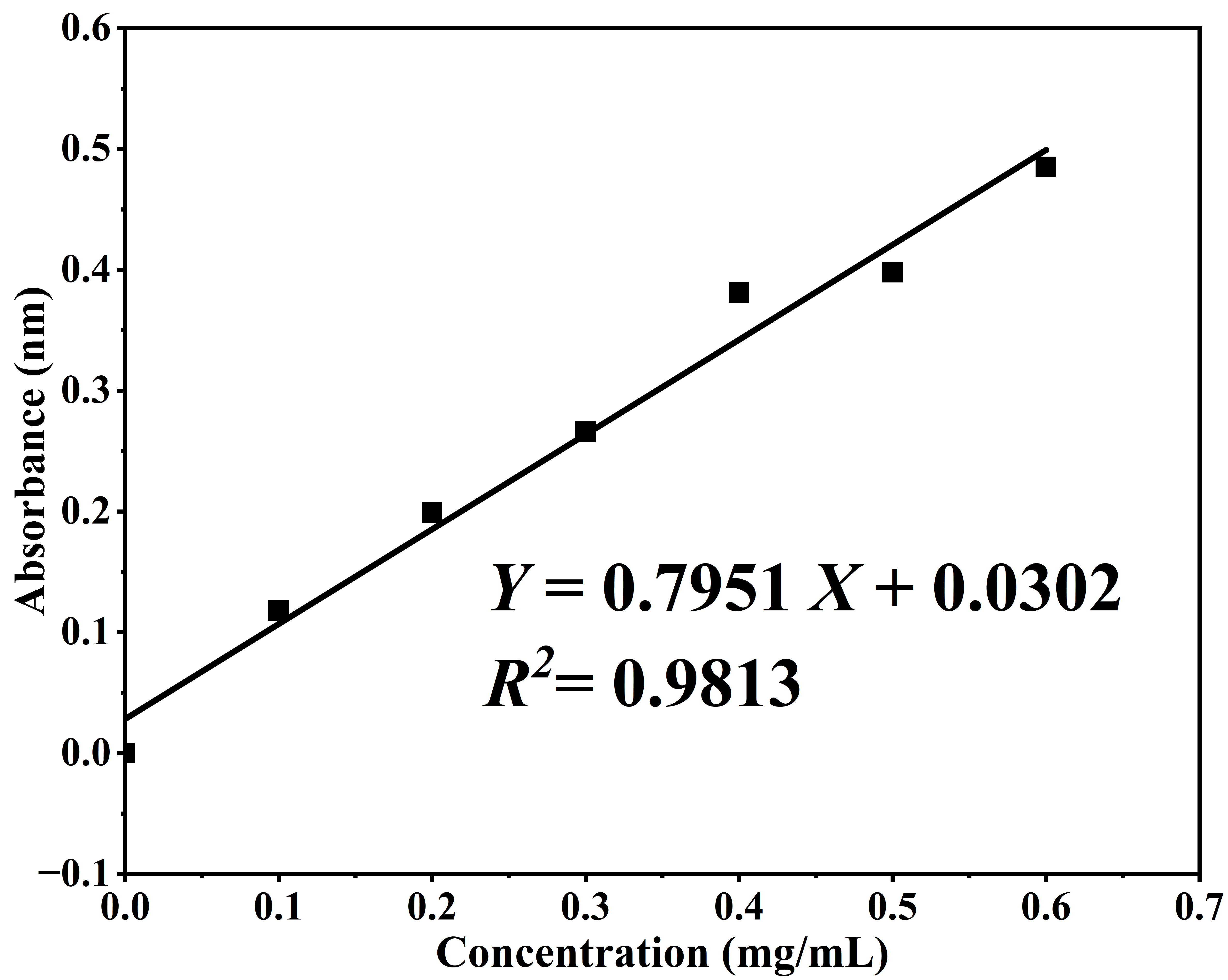
**

**Glucuronic acid standard curve:**

**
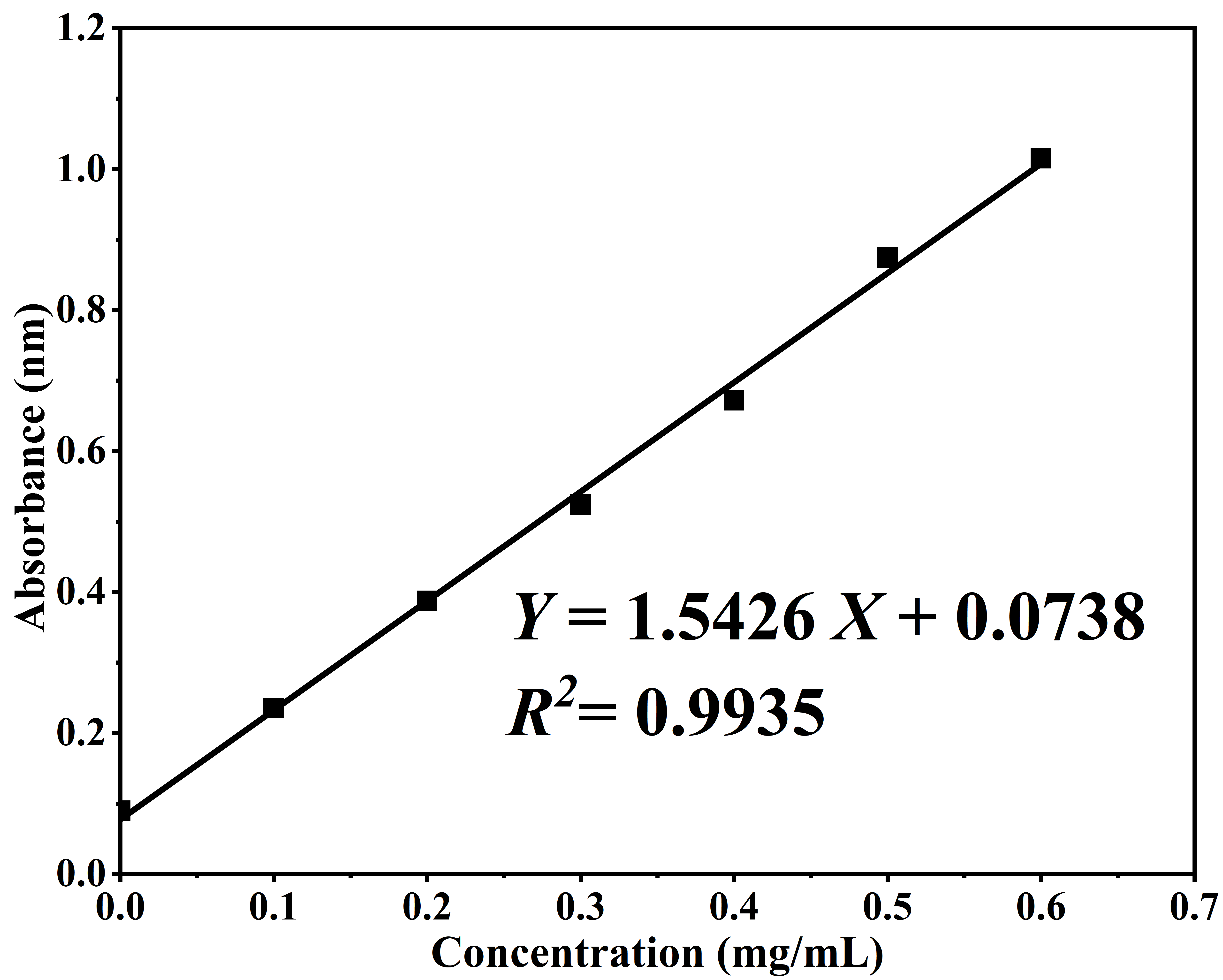
**
